# Discovery and preclinical evaluation of BPB-101: a novel triple functional bispecific antibody targeting GARP-TGF-β complex/SLC, free TGF-β and PD-L1

**DOI:** 10.3389/fimmu.2024.1479399

**Published:** 2024-11-20

**Authors:** Wenxin Xu, Jieying Xu, Pingcui Li, Deyu Xu, Hongjie Cheng, Huan Zheng, Li Zhang, Mengmeng Liu, Siyuan Ye, Mengshi Jiang, Wenqi Yu, Jiabing Wang, Lieming Ding

**Affiliations:** The R&D Department of Betta Biologic, Betta Pharmaceuticals Co. Ltd, Hangzhou, Zhejiang, China

**Keywords:** PD-1/PD-L1, TGF-β, GARP, GARP-TGF-β complex, small latent complex (SLC), Tregs, tumor microenvironment (TME), BPB-101

## Abstract

**Background:**

In the tumor microenvironment (TME), the transforming growth factor-β (TGF-β) and programmed cell death receptor 1 (PD-1)/programmed death ligand 1 (PD-L1) signaling axes are complementary, nonredundant immunosuppressive signaling pathways. Studies have revealed that active TGF-β is mainly released from the glycoprotein A repetitions predominant (GARP)-TGF-β complex on the surface of activated regulatory T cells (Tregs), B cells, natural killer (NK) cells, and tumor cells. The currently available antibodies or fusion proteins that target TGF-β are limited in their abilities to simultaneously block TGF-β release and neutralize active TGF-β in the TME, thus limiting their antitumor effects.

**Methods:**

We designed and constructed a bispecific, trifunctional antibody, namely, BPB-101, that specifically targets the GARP-TGF-β complex and/or small latent complex (SLC), active TGF-β, and PD-L1. The binding ability of BPB-101 to the different antigens was determined by ELISA, FACS, and biolayer interferometry (BLI). The blocking ability of BPB-101 to the TGF-β and PD-1/PD-L1 signaling axes was determined by reporter gene assay (RGA). The antitumor effect and biosafety of BPB-101 were determined in a transgenic mouse tumor model and cynomolgus monkeys, respectively. Stability assessments, including stability in serum, after exposure to light, after repeated freeze-thaw cycles, and after high-temperature stress tests had been completed to evaluate the stability of BPB-101.

**Results:**

BPB-101 bound efficiently to different antigenic proteins: the GARP-TGF-β complex and/or SLC, active TGF-β, and PD-L1. Data showed that BPB-101 not only effectively inhibited the release of TGF-β from human Tregs, but also blocked both the TGF-β and PD-1/PD-L1 signaling pathways. In an MC38-hPD-L1 tumor-bearing C57BL/6-hGARP mouse model, BPB-101 at a dose of 5 mg/kg significantly inhibited tumor growth, with a complete elimination rate of 50%. Stability assessments confirmed the robustness of BPB-101. Furthermore, BPB-101 showed a favorable safety profile in nonhuman primate (NHP) toxicity studies.

**Conclusion:**

BPB-101 is a potentially promising therapeutic candidate that may address unmet clinical needs in cancer immunotherapy, thus, BPB-101 warrants further clinical investigation.

## Introduction

Immunotherapy is radically altering the consistently poor prognosis of cancer patients by triggering long-term durable remission in certain patients (1, 2). Moreover, immunotherapy is playing a revolutionary role in cancer treatment and has reached a critical point, especially with the clinical application of immune checkpoint inhibitors (ICIs) and CAR-T cell therapies. Despite the great promise of ICIs, which include anti-programmed cell death receptor 1 (PD-1) antibodies, anti-programmed death ligand 1 (PD-L1) antibodies, and anti-cytotoxic T-lymphocyte-associated protein 4 (CTLA-4) antibodies, only approximately 20~30% of patients benefit from ICIs (3, 4). Scientists have aimed to explore the factors that may limit ICIs’ efficacy. One of the most important of these factors is the transforming growth factor-β (TGF-β). TGF-β has been suspected to play a key role in regulating the efficacy of cancer immunotherapy and has gradually become a focus of this research field (5, 6). Many studies have revealed that the TGF-β signaling axis is one of the main factors that lead to the development of tumor resistance to immunotherapy. Eduard Batlle reported that TGF-β in the tumor microenvironment (TME) determines T-cell exclusion and poor tumor response to ICIs (6). In addition, TGF-β in the TME also directly or indirectly suppresses the activity of innate immune cells, such as natural killer cells (NKs) and dendritic cells (DCs), by inhibiting the expression of the NKG2D ligand or interfering with antigen presentation (7, 8). Therefore, reducing the level of TGF-β in the TME has become a key goal (9, 10).

The production of TGF-β is a precisely regulated process. TGF-β is initially produced by immune cells, primary regulatory T cells (Tregs) and tumor cells in an inactive form (11). In the endoplasmic reticulum, following cleavage by the endoprotease Furin, dimerized latent TGF-β molecules form a hairpin small latent complex (SLC), which conceals the sites by which TGF-β binds to its receptor (12, 13). To date, the SLC has been shown to release active TGF-β through three pathways. The glycoprotein A repetitions predominant (GARP)-TGF-β pathway is one of the main pathways by which active TGF-β is released. GARP, which is a type I transmembrane cell surface docking receptor for the SLC, cooperates with αV integrins (GARP/αV) to release active TGF-β from the surface of Tregs, B cells, NK cells, platelets, cancer-associated fibroblasts (CAFs) and tumor cells, thereby promoting immunosuppressive functions of the TME (14).

Several GARP-targeting strategies have already shown some promising results, further inspiring research enthusiasm for this topic (15, 16). However, studies show that the GARP/αV integrin is the main pathway but not the only pathway involved in the production of active TGF-β. Active TGF-β can also be generated by the direct cleavage of the SLC by extracellular proteases or through the latent TGF-β binding protein (LTBP)/extracellular matrix (ECM)/αVβ6 axis (17, 18). Interference with the binding of the GARP-TGF-β complex has no apparent effect on the production of active TGF-β via other pathways or on active TGF-β that is already present in the TME. Thus, to effectively decrease the TGF-β level in the TME, it is necessary to neutralize the active TGF-β that are already present while also preventing the production of more active TGF-β molecules via a two-pronged approach.

Numerous studies have revealed the close connection between TGF-β and PD-L1, both of which are important components related to immune evasion (19, 20). Surprisingly, the inhibition of TGF-β is likely to induce the overexpression of PD-1 by tumor infiltrating lymphocytes (TILs), contributing to tumor cell survival (21). Active TGF-β promotes the production of exosomes with high levels of PD-L1 by cancer cells, and these exosomes mediate T-cell dysfunction via the early phosphorylation of T cell receptor (TCR) signaling domain. Moreover, TGF-β has been shown to be a drive of tumor tolerance to anti-PD-L1 immunotherapy (22, 23). In the TME, the PD-1/PD-L1 and TGF-β signaling pathways are overlapping but nonredundant pathways promote tumor survival. Thus, the suppression of TGF-β or PD-L1 alone has limited therapeutic efficacy (24, 25). Preclinical studies have shown that TGF-β inhibition combined with PD-L1 blockade has a significant synergistic effect that far exceeds that of either single treatment alone (26, 27). This combination strategy is the focus of current clinical investigations.

To mitigate the negative impact of TGF-β in the TME and thereby enhance the therapeutic effects of immunotherapy, our group designed and developed a trifunctional bispecific antibody (BPB-101) that targets both the GARP-TGF-β and the PD-1/PD-L1 signaling axes. Unlike existing PD-(L)1/TGF-β(R) therapeutic molecules or anti-GARP monoclonal antibodies, BPB-101 was designed to simultaneously target GARP-TGF-β complex or the SLC, active TGF-β, and PD-L1 on the basis that simultaneous inhibition of these pathways may be more effective for enhancing antitumor activity than inhibition of any one pathway alone.

Herein, we describe the discovery and preclinical assessment of BPB-101, including *in vitro* cytological experiments, *in vivo* antitumor studies and the safety profile analyses in nonhuman primates (NHPs).

## Materials and methods

### Materials

The list of materials used in this study is available in the **Supplementary Materials** and **Supplementary Methods** in **Supplementary Data Sheet 1**.

### Cell lines and animals

The information of cells lines and animals in this study is available in the **Supplementary Materials** and **Supplementary Methods** in **Supplementary Data Sheet 1**.

### Purify analysis of BPB-101

BPB-101, BPB-GARP and BPB-PD-L1 were expressed in Expi293F cells grown in shake flasks. The cell culture supernatants were harvested by centrifugation and then passed over protein A agarose (MabSelect SuReTM from Cytiva). Bound antibodies were washed with buffer at pH 3.4 (**Supplementary Methods** in **Supplementary Data Sheet 1**). To further purify the antibodies and remove aggregates and fragments, cation exchange chromatography (CEX) was employed. The protein solution was adjusted to pH 5.0, and the CEX resin was equilibrated with 50 mM acetate (Sigma). Elution was performed using a linear gradient, and the peak fractions were collected. The samples were then analyzed for purity using size-exclusion chromatography (SEC) (**Supplementary Methods** in **Supplementary Data Sheet 1**).

### Binding of BPB-101 to GARP-TGF-β complex, active TGF-β or PD-L1

Briefly, 293F-GARP-TGF-β (4E9) or 293T-hPD-L1 cells were plated in 96-well plates and incubated with antibodies, followed by FACS analysis. In addition, human PD-L1 protein, human GARP-TGF-β complex, or human TGF-β protein was coated onto 96-well plates and incubated overnight. Antibodies were then added to the plates, followed by the addition of goat anti-human IgG-Fc-HRP. The biotin signal value was measured at 450 nm (**Supplementary Methods** in **Supplementary Data Sheet 1**). For detailed information on the antigens and antibodies used, please refer to the **Supplementary Materials**.

### Biolayer interferometry

The binding kinetics were determined using an Octet RED96E system with anti-human Fc AHC sensors (Sartorius, 18-5064) or AHC2 sensors (Sartorius, 18-5142) to capture antibodies (28, 29). After establishing a 120-second baseline step in SD buffer, a 1:1 serial dilution of the human antigen in SD buffer was performed. Subsequently, the binding and dissociation processes of the antibody and antigen were detected (**Supplementary Methods** in **Supplementary Data Sheet 1**). For detailed information about the antigens and antibodies used, please refer to the **Supplementary Materials**.

### Competition binding assays of BPB-101 with PD-L1 and CD80

293T-hPD-1 and 293T-hPD-L1 cells were plated into 96-well plates and cultured with a mixture containing antibodies, biotin-conjugated PD-L1 protein, or CD80-mFc protein. One hour later, the cells were centrifuged using an Eppendorf 5810R centrifuge and washed twice with FACS buffer. Following the addition of SA-PE or R-PE-goat anti-mouse IgG Fc, the cells were analyzed with flow cytometry.

### Luciferase reporter gene assay

To verify the blocking effect of BPB-101 on the downstream signaling of TGF-β and PD-L1, two reporter systems were utilized. The 293T-hPD-L1 and 293-TGF-β/GARP-αvβ6 (4D11) cells were used as upstream cells to provide hPD-L1 or TGF-β protein, respectively. Jurkat-PD-1-CD3zeta-NFAT-Luc2 and 293-SBE-res (1E9) cells were used as effector cells to generate a fluorescent signal in response to the upstream signaling. This signal was detected in conjunction with the One-Glo reagent (**Supplementary Methods** in **Supplementary Data Sheet 1**).

### Allogeneic mixed lymphocyte reaction experiment

PBMCs were resuspended in RPMI 1640 culture medium and adjusted to a concentration of 1E6 cells/mL, as measured using a BECKMAN VI-CELL XR. Mature DCs from another donor were resuspended at a concentration of 2E5 cells/mL. Then, 100 μL of PBMCs, 50 μL of DCs and 50 μL of antibodies were mixed well in 96-well plates. The mixture was cultured at 37°C for 5 days and then analyzed using an ELISA kit (**Supplementary Methods** in **Supplementary Data Sheet 1**).

### Cytokine secretion of Tregs

The anti-human CD3 and anti-human CD28 (1 μg/mL) were coated onto 96-well plates and cultured at 4°C overnight. The plates were washed three times with DPBS. Tregs, cultured in RPMI 1640 containing 500 IU/mL recombinant human IL-2 (rhIL-2), were added to the plates at a concentration of 4E5 cells/well (**Supplementary Methods** in **Supplementary Data Sheet 1**). After five days, the supernatants were collected, and the TGF-β levels were measured using an ELISA kit. For detailed information about the antibodies used, please refer to the **Supplementary Materials**.

### Binding of BPB-101 to different immune cells in the blood

Red blood cells (RBCs) were removed from human blood samples using an RBC lysis buffer (BioLegend, 420302). Then, the FCR blocking reagent (Miltenyi, 130-059-901) was applied to block nonspecific binding sites on the immune cells. BPB-101 and hIgG1 were serially diluted in FACS buffer, and their binding to CD4^+^ T cells, CD8^+^ T cells, Tregs, pDCs, classical monocyte cells, nonclassical monocyte cells, B cells (CD19^+^), NK cells and NKT cells was determined by FACS (30–32). For detailed information about of the antibodies used, please refer to the **Supplementary Materials**.

### Mouse studies

All *in vivo* work was approved by the Jiangsu Provincial Department of Science and Technology Animal Control Committee (AP-MIJ220068). The main experiments focused on investigating the biodistribution and antitumor activity of BPB-101 (**Supplementary Methods** in **Supplementary Data Sheet 1**).

### Statistics

Data are presented as the mean ± standard error of the mean (SEM) unless otherwise indicated. Statistical significance was analyzed by the unpaired two-tailed Student’s t test. P values below 0.05 were considered to indicate statistical significance (* *P* < 0.05, ** *P* < 0.01, *** *P* < 0.001, **** *P* < 0.0001, ns: not significant).

## Results

### Screening, construction, and purification of BPB-101

**Figure 1:**
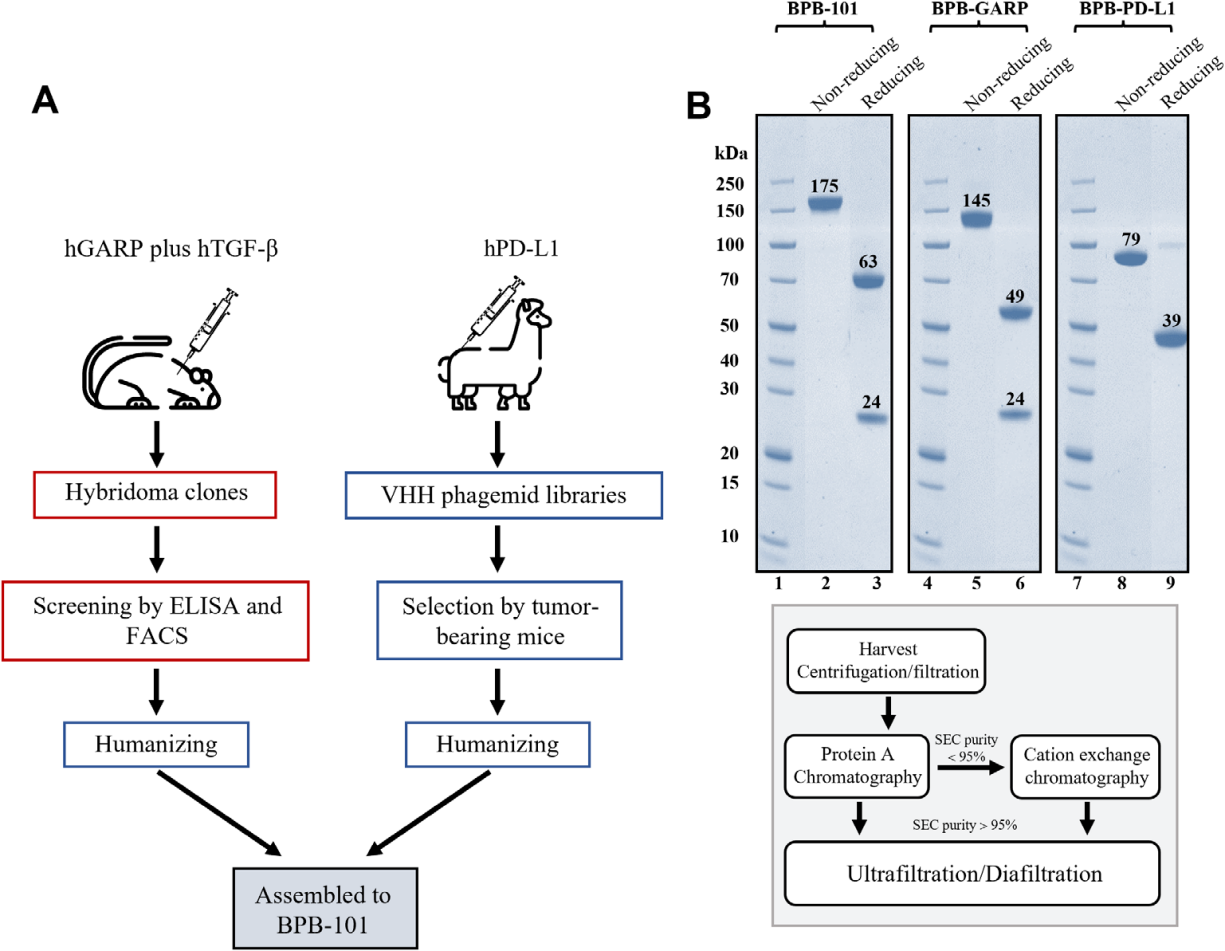
Screening, construction and purification of BPB-101. **(A)**, A simple schematic flow chart illustrates the construction and screening process of BPB-101. **(B)**, SDS-PAGE analysis of purified BPB-101, BPB-GARP (a parental mAb targeting GARP) and BPB-PD-L1 (a parental mAb targeting PD-L1) (upper panel). Schematic showing the purification of BPB-101, BPB-GARP and BPB-PD-L1 (lower panel). Lanes 1,4, and 7: the same marker, 2: BPB-101 in the nonreducing form, 3: BPB-101 in the reducing form, 5: BPB-GARP in the nonreducing form, 6: BPB-GARP in the reducing form, 8: BPB-PD-L1 in the nonreducing form, 9: BPB-PD-L1 in the reducing form.

An anti-GARP monoclonal antibody (mAb) was discovered using a hybridoma platform and further selected for its binding to the GARP-TGF-β complex, the SLC and active TGF-β. An anti-PD-L1 nanoantibody (nAb) was screened from a VHH library and then optimized in tumor-bearing mice. Subsequently, BPB-101was successfully generated by combining the intact heavy and light chains of the humanized anti-GARP antibody with the humanized anti-PD-L1 nAb (VHH). The VHH domain is located at the C-terminus of BPB-101 (**Figure 1A**). The sequence of BPB-101, which includes VH, VL and VHH, differs from that of other mAbs associated with GARP, TGF-β and PD-L1. The Fab arm of the anti-GARP component differs from the VH and VL regions of reference antibodies, with the greatest variability in the complementarity-determining regions (CDR)-H3 and CDR-L3. Furthermore, the CDRs (PD-L1-binding domains) of M7824 (a PD-L1/TGF-βRII inhibitor) and atezolizumab (a PD-L1 inhibitor) were compared with the C-terminus (a PD-L1 inhibitor) of BPB-101. The VHH sequence of BPB-101 significantly differs from those of atezolizumab and M7824, with sequence similarities of 72.88% and 72.50%, respectively. All these results indicate that BPB-101 has a unique amino acid sequence (**Supplementary Table S1**, **Supplementary Figure S1**). For subsequent studies, BPB-101 was further purified using protein A agarose resin. After affinity chromatography, BPB-101 was subjected to sodium dodecyl sulfate-polyacrylamide gel electrophoresis (SDS-PAGE), and no bands indicating impurities were observed (**Figure 1B**).

### BPB-101 is a unique trifunctional antibody that binds efficiently to the GARP-TGF-β complex, active TGF-β and PD-L1

**Figure 2:**
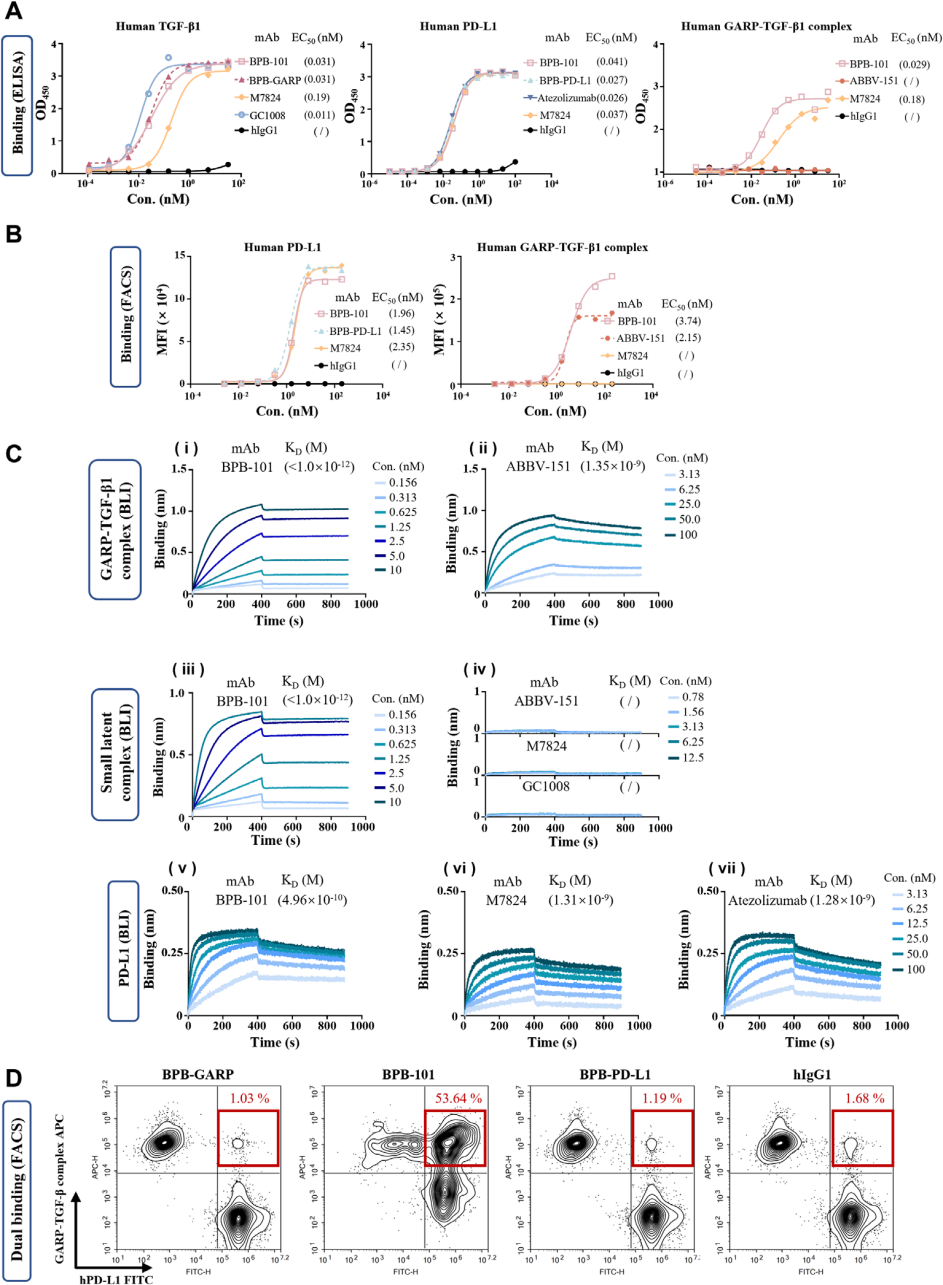
BPB-101 bound efficiently to human TGF-β, human PD-L1 and the human GARP-TGF-β1 complex. **(A)** Binding of different antibodies to human TGF-β, human PD-L1 or the human GARP-TGF-β1 complex as determined by ELISA. **(B)** Binding of different antibodies to 293T-GARP-TGF-β1 complex cells (4E9) or 293T-hPD-L1 cells as detected by FACS. **(C)** (i and ii) Biolayer interferometry (BLI) results showing the interaction of the GARP-TGF-β1 complex with immobilized BPB-101 and ABBV-151 (a GARP-TGF-β1 complex inhibitor) at 10 μg/mL. The concentrations of the GARP-TGF-β1 complex ranged from 0.156 to 10 nM for BPB-101 and from 3.13 to 100 nM for the other assays. The dissociation rate of BPB-101 was less than 1 x 10^-7^ 1/s, and it took more than 14 hours to achieve 5% dissociation; thus, an accurate K_D_ could not be calculated (see **Supplementary Table S1** for details). (iii and iv) BLI analysis of the SLC with immobilized BPB-101, ABBV-15, M7824 (PD-L1/TGF-βRII inhibitor) and GC1008 (TGF-β1/2/3 inhibitor) at 10 μg/mL. The concentrations of the SLC ranged from 0.156 to 10 nM for BPB-101 and ranged from 0.78 to 12.5 nM for the other assays. The binding of ABBV-151, M7824 and GC1008 to SLC was not detectable. (v - vii) The binding of human PD-L1 with immobilized M7824 and atezolizumab (PD-L1 inhibitor) at 10 μg/mL as detected by BLI. The concentrations of PD-L1 ranged from 3.13 to 100 nM for each assay. The dissociation constant expressed by K_D_ was determined from the detailed binding traces of different mAbs. **(D)** Dual binding ability of BPB-GARP, BPB-101, BPB-PD-L1 and hIgG1 at 0.8 nM with CSFE-labeled 293T-hPD-L1 cells and FarRed-labeled 293F-GARP-TGF-β (4E9) cells.

There are three TGF-β isoforms: TGF-β1, TGF-β2, and TGF-β3. The binding of active TGF-β to the corresponding receptor leads to the phosphorylation and activation of the canonical signaling molecules SMAD2 and SMAD3. We examined the binding of BPB-101 to these three TGF-β isoforms and found that the binding of BPB-101 to human TGF-β2 and TGF-β3 was relatively weak compared to its binding to human TGF-β1 (**Figure 2A**, **Supplementary Figures S2A, B**). Therefore, TGF-β1 was selected for follow-up studies to test BPB-101’s ability to block TGF-β and terminate TGF-β-dependent signaling. The ELISA results in **Figure 2A** (1^st^ panel) suggested that BPB-101 has a strong avidity for active human TGF-β1, with an EC_50_ of 0.031 nM; which was superior to that of M7824 (EC_50_ = 0.19 nM). Moreover, the DS-1055a (a GARP inhibitor) and ABBV-151 (a GARP-TGF-β1 complex inhibitor) both failed to bind to human active TGF-β1 (**Supplementary Figure S2C**). The binding of BPB-101 to human PD-L1 is another important precondition of its biological activity. BPB-101, BPB-PD-L1 (the parental antibody of BPB-101), M7824, and atezolizumab bound to PD-L1 with similar EC_50_ values ranging from 0.02 to 0.04 nM (**Figure 2A**, 2^nd^ panel). BPB-101’s binding ability to the human GARP-TGF-β1 complex, with an EC_50_ of 0.029 nM, was stronger than that of the positive controls (**Figure 2A**, 3^rd^ panel). Interestingly, in this study, we did not detect binding activity of ABBV-151, which could theoretically bind to the GARP-TGF-β complex. However, M7824, which theoretically does not bind to the GARP-TGF-β complex, produced a binding curve with an EC_50_ of 0.18 nM. This result likely occurred due to a change in the conformation of the GARP-TGF-β complex antigen, as this phenomenon did not occur in subsequent cell-based binding experiments. The FACS data showed that the EC_50_ values of BPB-101 in 293T-GARP-TGF-β1 complex cells (4E9) and 293T-hPD-L1 cells were 3.74 nM and 1.96 nM, respectively; these values were higher than those of M7824, which could not bind to 4E9 cells at all (**Figure 2B**). In conclusion, we confirmed that BPB-101 binds specifically to human TGF-β1, PD-L1, and the GARP-TGF-β1 complex in a dose-dependent manner.

We evaluated the avidity of antibodies for the GARP-TGF-β1 complex, the SLC and PD-L1 using biolayer interferometry (BLI) (**Figure 2C**, **Supplementary Table S2**). The dissociation constant (K_D_) of BPB-101 for the GARP-TGF-β1 complex was less than 1 x 10^-12^ M due to its slow dissociation rate, and the avidity of the ABBV-151 control antibody for the GARP-TGF-β1 complex was nearly 3 orders of magnitude lower than that of BPB-101 (**Figures 2C**i, ii, **Supplementary Table S2**). For the SLC, BPB-101 yielded a K_D_ of <1 x 10^-12^ M, indicating a high binding efficiency. The remaining control antibodies-namely, ABBV-15, M7824 and GC1008 (a TGF-β1/2/3 inhibitor)-did not bind to the SLC (**Figures 2C**iii, iv, **Supplementary Table S2**). The significantly lower k_off_ value of BPB-101 indicated that it stably bound to the GARP-TGF-β1 complex or the SLC and was unlikely to dissociate after binding to these antigens. As expected, BPB-101 exhibited high avidity for PD-L1, with a K_D_ of 4.96 x 10^-10^ M; this value was approximately 3-fold lower than those of M7824 and atezolizumab (**Figures 2C**v–vii, **Supplementary Table S2**). Next, we evaluated BPB-101’s ability to simultaneously bind to human PD-L1-expressing cells and human GARP-TGF-β1 complex-expressing cells. The percentage of double-positive cells in these populations tended to increase as the BPB-101 concentration increased from 0.00128 nM to 0.8 nM. At BPB-101 concentrations higher than 0.8 nM (4 nM, 20 nM and 100 nM), the dual binding of BPB-101 to the two cell lines became saturated (**Figure 2D**, **Supplementary Figure S2D**); these results suggested that BPB-101 has robust dual-target specificity.

### BPB-101 can simultaneously block the TGF-β and PD-L1 pathways and their downstream signals

**Figure 3:**
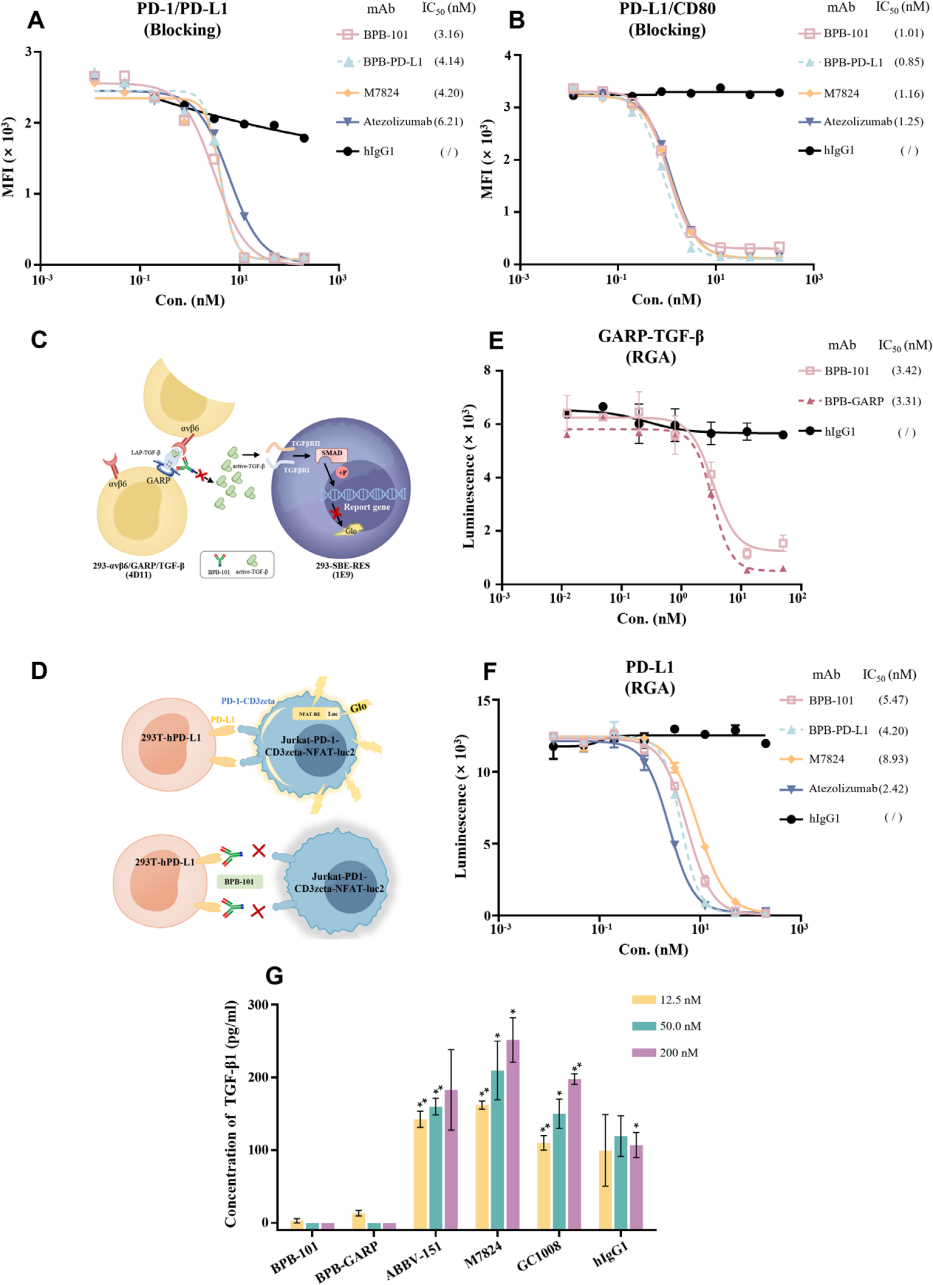
BPB-101 blocked the binding of PD-L1 to PD-1 or CD80 and the transduction of TGF-β and PD-L1 signals. **(A, B)** Abilities of BPB-101, BPB-PD-L1, M7824, atezolizumab and hIgG1 to block PD-1/PD-L1 and PD-L1/CD80 as detected by ELISA. **(C)** Schematic diagram of RGA of BPB-101 in blocking GARP-TGF-β signaling. **(D)** Schematic diagram of RGA of BPB-101 in blocking PD-1/PD-L1 signaling. **(E)** Luminescence results of 293-SBE-RES cells in RGA. **(F)** Luminescence results of Jurkat-PD1-CD3zeta-NFAT-luc2 cells in RGA. **(G)** The concentrations of TGF-β1 in the supernatants of Tregs cocultured with BPB-101, BPB-GARP, ABBV-151, M7824, GC1008 or hIgG1 (12.5 nM, 50 nM or 200 nM) for 5 days were detected by ELISA. The data are expressed as mean ± SEM. P values were calculated (different mAbs vs. BPB-101 at the same concentration). The statistical analyses were performed using unpaired two-tailed Student’s t tests. (*P < 0.05, **P < 0.01, ns, not significant, not shown).

It has been reported that, in addition to binding to PD-1, PD-L1 also binds to CD80 in its receptor form. When the PD-1/PD-L1 and CD80/PD-L1 pathways are activated, the ability of T cells to kill tumor cells is impaired (33, 34). Here, we assessed the ability of BPB-101 to target and block PD-L1, using atezolizumab and M7824 as controls. As expected, BPB-101 blocked the binding of PD-1/PD-L1 (**Figure 3A**) and CD80/PD-L1 (**Figure 3B**) interactions in a dose-dependent manner, with IC_50_ values of 3.16 nM and 1.01 nM, respectively; these values were significantly lower than those of M7824 and atezolizumab. Luciferase reporter gene assays were then used to further evaluate the inhibitory effect of BPB-101 on TGF-β and PD-L1 signal transduction. Two cell line systems were established for this assay: 1) HEK-293T cells expressing TGF-β-GARP-αvβ;6 and 293-SBE-RES cells (**Figure 3C**) and 2) HEK-293T cells expressing PD-L1 and a luciferase reporter driven by a native response element, namely PD-L1 effector cells (Jurkat-PD-1-CD3zeta-NFAT-luc2) (**Figure 3D**). These two systems were co-cultured in the presence of different mAbs, and the degree to which the downstream effector cells were activated was determined by measuring the luciferase activity. BPB-101 and BPB-GARP obviously and effectively reduced the fluorescence signal intensity of the 293-SBE-RES cells (**Figure 3E**, **Supplementary Figure S3**), and the fluorescence signal intensity of the PD-L1 effector cells was decreased by BPB-101 and BPB-PD-L1. Moreover, in this system, the blocking ability of BPB-101 was weaker than that of atezolizumab and BPB-PD-L1, but stronger than that of M7824 (**Figure 3F**). The decreased in the downstream fluorescence signal demonstrated the strong ability of our antibodies to inhibit these two immunosuppressive signals. In addition, we evaluated the impact of our mAbs on TGF-β production. The abundant expression of GARP on the surface of Tregs is the main cause of the release of active TGF-β (35). When BPB-101 bound to the GARP-TGF-β complex on Tregs, it effectively prevented the secretion of TGF-β. Tregs were incubated with antibodies for 5 days, after which the TGF-β levels in the supernatants were measured by ELISA. We confirmed that BPB-GARP and BPB-101, but not ABBV-151, GC1008, M7824 or BPB-PD-L1, inhibited TGF-β release by Tregs (**Figure 3G**, **Supplementary Figure S4**).

### BPB-101 effectively enhances IFN-γ secretion by PBMCs

**Figure 4:**
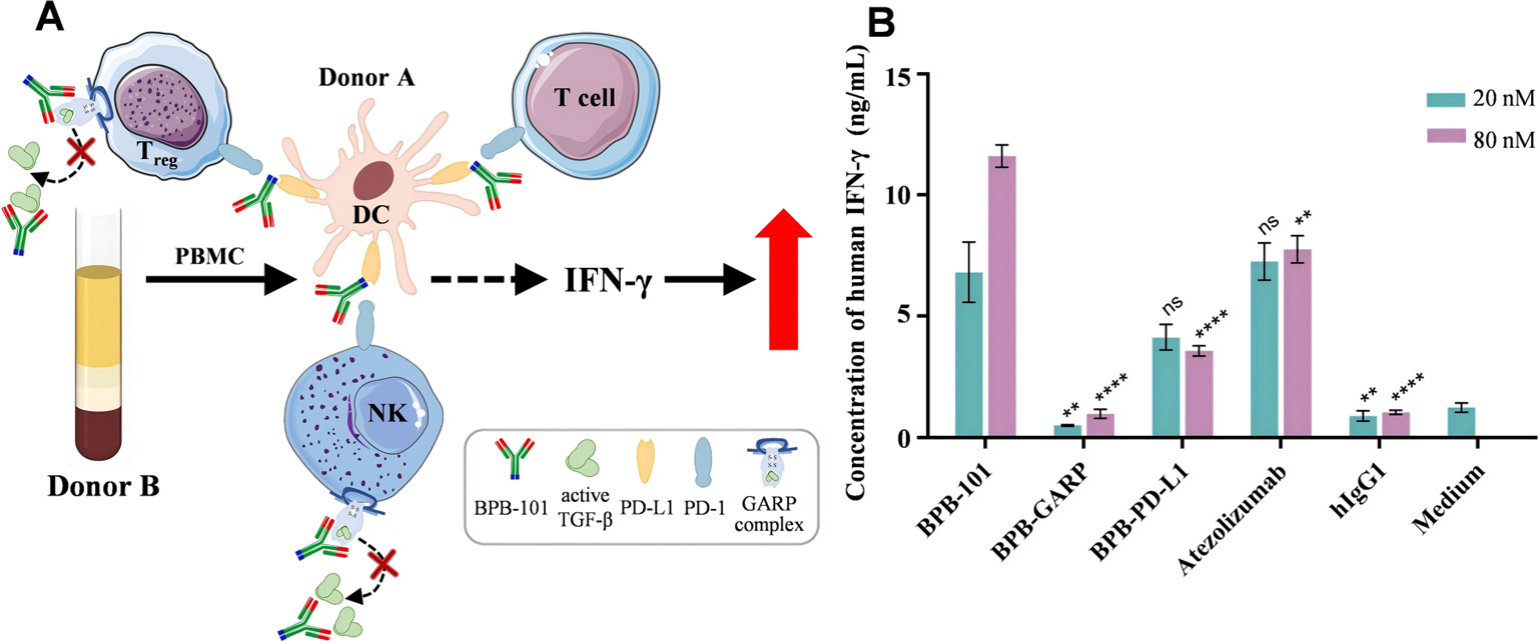
BPB-101 increased IFN-γ secretion by PBMCs. **(A)** Schematic diagram of the allogeneic mixed lymphocyte reaction assay. The anti-PD-L1 component is illustrated in blue at the C-terminus of BPB-101, while the Fab region, depicted in red, represents the anti-GARP complex. **(B)** PBMCs and DCs were cocultured with 20 nM or 80 nM BPB-GARP, BPB-PD-L1, BPB-101, atezolizumab or hIgG1 at for 5 days, and the concentrations of human IFN-γ in the supernatants were determined by ELISA. P values were calculated (different mAbs vs. BPB-101 at the same concentration). The data are expressed as the mean ± SEM. The statistical analyses were performed using unpaired two-tailed Student’s t tests. (**P < 0.01, ****P < 0.0001, ns, not significant, not shown).

The allogeneic mixed lymphocyte reaction (MLR) is an experimental approach in which a mixture of lymphocytes from two unrelated individuals is cultured together, stimulating each other due to the different histocompatibility antigens on their membranes. This leads to cell division and proliferation, as well as the transformation of each lymphocyte. DCs, a class of APCs, can stimulate the activation and proliferation of T cells and induce the secretion of human IFN-γ (hIFN-γ). DCs that express PD-L1, which binds to PD-1 on T cells, can deliver negative immune regulatory signals and inhibit the secretion of hIFN-γ. In addition, Tregs and NK cells derived from PBMCs secrete TGF-β, which in turn inhibits the secretion of hIFN-γ (**Figure 4A**) (36, 37). We previously showed that BPB-101 could specifically bind to PD-L1 and the GARP-TGF-β1 complex, thereby blocking the PD-1/PD-L1 signaling pathway and TGF-β secretion (**Figures 2A**, **3**). To verify whether BPB-101 relieves the associated immunosuppressive effects and promotes the secretion of hIFN-γ, we measured the auxiliary activating effects of BPB-101 on PBMCs in an MLR system. As expected, BPB-101, BPB-PD-L1 (a humanized monoclonal parental antibody) or atezolizumab treatment enhanced the activation of PBMCs in the presence of DCs, as indicated by an increased in the level of IFN-γ. At a concentration of 20 nM, the effects of BPB-101 and atezolizumab were similar, and both were slightly stronger than that of BPB-PD-L1. However, at a concentration of 80 nM, the effect of BPB-101 surpassed that of atezolizumab. The other humanized monoclonal parental antibody of BPB-101 (BPB-GARP) exerted almost no effect in this study system, even at high concentrations. Thus, the activity of the BPB-101 was superior to that of its constituent monoclonal antibodies (**Figure 4B**).

### Assessment of the potential *in vitro* side effects of BPB-101

**Figure 5:**
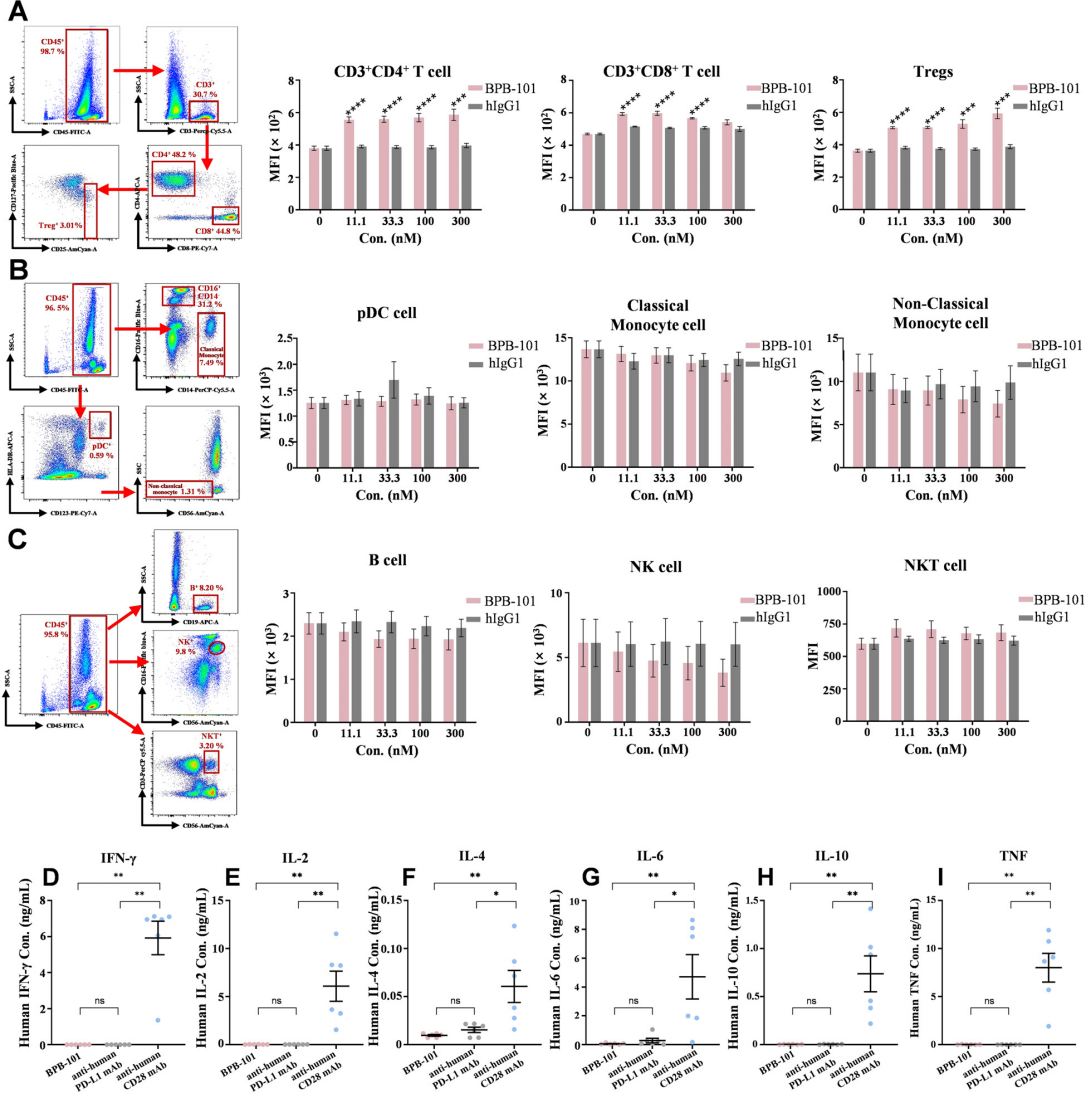
*In vitro* risk assessment of BPB-101. **(A)** FACS analysis of CD4^+^ T cells (CD3^+^CD4^+^), CD8^+^ T cells (CD3^+^CD8^+^) and Tregs (CD3^+^CD4^+^CD25^+^CD127^low^) in the blood. A representative sample is shown to illustrate the gating strategy. P values were calculated (BPB-101 vs. hIgG1 at the same concentration). **(B)** FACS analysis of pDCs (HLA-DR^+^CD123^+^), classical monocyte cells (CD14^+^CD16^-^) and nonclassical monocyte cells (CD16^+^CD56^-^CD14^-^) in the blood. A representative sample is shown to illustrate the gating strategy. **(C)** FACS analysis of B cells (CD19^+^), NK (CD56^+^CD16^+^) and NKT cells (CD3^+^CD56^+^) in the blood. A representative sample is shown to illustrate the gating strategy. All the values are presented as the mean values of three donors, and each experiment was repeated three times. An anti-human CD28 antibody (TGN1412) was used as a positive control, and an anti-human PD-L1 antibody (atezolizumab) was used as a negative control for the assessment of cytokine secretion by PBMCs. After incubation with three mAbs (10 μg/mL), the supernatants were collected, and the levels of IFN-γ **(D)**, IL-2 **(E)**, IL-4 **(F)**, IL-6 **(G)**, IL-10 **(H)** and TNF **(I)** in the supernatants were determined by FACS according to the experimental methods of the Cytometric Bead Array (CBA) Human Th1/Th2 Cytokine Kit II. PBMCs were obtained from six healthy donors. All the data are expressed as the mean ± SEM. The statistical analyses were performed using unpaired two-tailed Student’s t tests. (*P < 0.05, **P < 0.01, ***P < 0.001, ****P < 0.0001, ns, not significant).

To evaluate the potential side effects of BPB-101, whole blood samples from three healthy donors were incubated with different antibodies, and the binding of these antibodies to various immune cells was determined by FACS. The binding of BPB-101 to nine main types of immune cells was investigated. Compared with hIgG1, BPB-101 exhibited significantly greater binding to CD4^+^ T cells, CD8^+^ T cells and Tregs (**Figure 5A**), which was consistent with its outstanding ability to bind to the GARP-TGF-β complex and PD-L1 (**Figure 2A**). However, BPB-101 failed to bind to pDCs, classical monocyte cells, nonclassical monocyte cells, B cells, NK cells or NKT cells, all of which play key roles in immunoregulation (**Figures 5B, C**). On this basis, it is important to understand whether BPB-101 causes abnormal cytokine release, as antibody-induced cytokine release may induce fatal adverse effects, known as cytokine release syndrome (CRS), in the clinic (38, 39). Different mAbs were added to PBMCs, and the levels of IL-2, IL-4, IL-6, IL-10, TNF and IFN-γ were secreted by PBMCs were monitored to assess the potential side effects of these mAbs. As shown in **Figures 5D–I**, the effect of BPB-101 was quite similar to that of the negative control (atezolizumab, an anti-human PD-L1 antibody). In contrast, the reference antibody (TGN1412, an anti-human CD28 antibody) significantly stimulated the secretion of these cytokines by PBMCs. These findings suggested that BPB-101 could bind specifically to CD4^+^ T cells, CD8^+^ T cells and Tregs without eliciting CRS.

### BPB-101 has satisfactory tumor-targeting properties and antitumor effects

**Figure 6:**
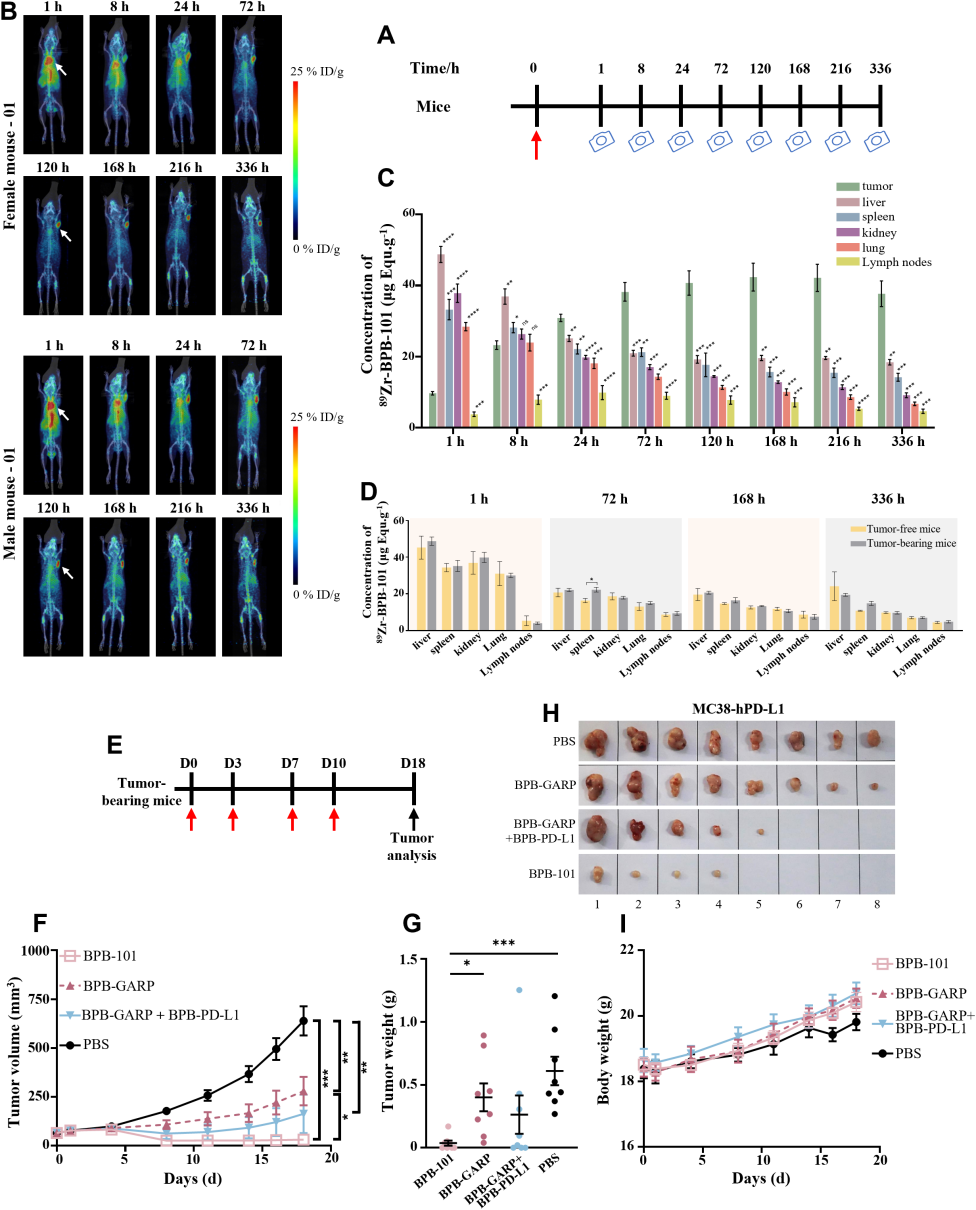
BPB-101 exhibited obvious tumor targeting ability and antitumor effects. **(A)** Schematic outline of the assay of BPB-101 biodistribution, red arrow: drug injection, camera: immune PET imaging. **(B)** Maximum intensity projection (MIP) slices obtained 1, 8, 24, 72, 120, 168, 216 and 336 hours after the administration of ^89^Zr-BPB-101 to tumor-bearing mice. (5 mg/kg, i.v., one dose). The white arrow indicates the tumor site. **(C)** Concentrations of ^89^Zr-BPB-101 in the tumors, livers, spleens, kidneys, lungs and lymph nodes of tumor-bearing mice at different time points (n = 4). **(D)** Biodistribution data of ^89^Zr-BPB-101 in the livers, spleens, kidneys, lungs and lymph nodes of tumor-bearing mice (n = 4) and tumor-free mice (n = 2) at 1 h, 72 h, 168 h and 336 h, respectively. P values were calculated (tumor-bearing mice vs tumor-free mice at the same time). **(E)** Schematic outline of the antitumor experiment; red arrow: drug injection, black arrow: tumor analysis. **(F)** Tumor-bearing mice were treated with BPB-101, BPB-GARP, BPB-101 + BPB-PD-L1 or PBS, and the tumor volume was recorded (5 mg/kg, i.p., four doses, n = 8). The P value shown on the top of the column is the comparison of tumor volume on D18. The results are presented as the mean ± SEM. **(G)** Average tumor weight of different mAb-treated mice on D18. **(H)** Images of tumors in different groups on D18 (the source data are shown in **Supplementary Figure S6**). **(I)** Changes in body weight of mice in all the groups. All the data are expressed as the mean ± SEM. The statistical analyses were performed using unpaired two-tailed Student’s t tests. (*P < 0.05, **P < 0.01, ***P < 0.01, ****P < 0.0001, ns, not significant, not shown).

According to the previous results of species cross-reaction experiment, BPB-101 can only bind to PD-L1 and GARP-TGF-β complex proteins of human and cynomolgus monkeys, but not to PD-L1 and GARP-TGF-β complex proteins of mice. Therefore, in the subsequent *in vivo* experiments, transgenic mice and cynomolgus monkeys were used as relevant species, respectively. The tumor-targeting ability of BPB-101 was explored in C57BL/6-hGARP mice bearing MC38-hPD-L1 tumors, with healthy C57BL/6-hGARP mice serving as controls. BPB-101 was first labeled with ^89^Zr according to a previously reported method (40, 41). ^89^Zr-BPB-101 was then intravenously administered, and the mice were scanned via PET/CT at preset times (**Figure 6A**). The radioactivity per unit volume of tumors, livers, spleens, kidneys, lungs and lymph nodes was analyzed by PMOD software. The percentage injection dose per gram of tissue (%ID/g) was calculated based on the administration dose. Once injected, the radiation of ^89^Zr-BPB-101 was immediately distributed in the tumors of tumor-bearing female or male mice, and the amount of mAbs in the tumor gradually increased over time. Twenty-four hours later, the radiation signal of the drug in tumors exceeded that in livers, and the accumulation of the drug in the tumors was consistently greater than that in any other tissue. A high concentration of ^89^Zr-BPB-101 remained in the tumor until 336 h, suggesting that BPB-101 gradually accumulated in the tumor (**Figures 6B, C**). However, in both healthy female and male mice, ^89^Zr-BPB-101 was concentrated in livers (**Supplementary Figures S5A, B**). We compared the distribution of BPB-101 in a series of tissues collected from tumor-bearing mice and tumor-free healthy mice. **Figure 6D** shows that there was a statistically significant difference in drug accumulation in the spleen between normal mice and tumor-bearing mice at 72 h. No statistically significant differences in drug distribution were noted at other time points or in other tissues. These data indicated that BPB-101 had satisfactory tumor-targeting ability. Moreover, the tumor volume of mice treated with ^89^Zr-BPB-101 tended to decrease over time, indicating that BPB-101 has a potential inhibitory effect on tumor growth (**Supplementary Figure S5C**).

The antitumor effect of BPB-101 was more pronounced in subsequent trials. C57BL/6-hGARP mice bearing MC38-hPD-L1 tumors were randomly divided into four groups and intraperitoneally injected with PBS, BPB-GARP (4.2 mg/kg), BPB-GARP + BPB-PD-L1 (4.2 + 2.3 mg/kg), or BPB-101 (5 mg/kg) twice a week for a total of four injections (**Figure 6E**). The changes in tumor volume and body weight of the mice were recorded every other day. Throughout the experiment, the tumor volume and tumor weight of the mice in the BPB-101 group decreased, and at the end of the experiment, the tumors in half of the mice (4/8) had completely disappeared (**Figures 6F–H**, **Supplementary Figure S6**). The average tumor volume of the mice in the BPB-101 group remained below 50 mm^3^ at the end of the experiment. In contrast, the tumor volume of the mice in the PBS group continued to increase, eventually reaching approximately 700 mm^3^ (**Figure 6F**). There were significant differences in tumor volume and tumor weight between the BPB-101 group and the PBS group on D18 (**Figures 6F, G** **).** Although the tumor volume in the BPB-GARP + BPB-PD-L1 group was significantly lower than that in the PBS group, and the tumor elimination rate reached 37.5% (3/8), there was no significant difference in tumor weight (**Figure 6G**). Additionally, the tumor volume in the BPB-GARP group was significantly lower than that in the PBS group. Compared to those in the BPB-GARP group, the tumor volume and tumor weight of the mice in the BPB-101 group decreased significantly (**Figures 6F, G**). The observed antitumor activity of BPB-101 was encouraging, and there was no evidence of tumor recurrence in our study, suggesting the potential of BPB-101 to sustain the immune response. The body weight of the mice increased slowly in all groups, and no visible toxic side effects were observed (**Figure 6I**).

### Evaluation of BPB-101 safety *in vivo*

To further evaluate the safety of BPB-101, a preclinical NHP toxicity study was conducted in cynomolgus monkeys. The cynomolgus monkeys were intravenously injected with 30 mg/kg or 100 mg/kg BPB-101 in a volume of 15 mL/kg, and control monkeys were injected with the same amount of solvent (15 mL/kg). The levels of IL-2, IL-6, TNF-α and IFN-γ in the serum were measured at preset times. Data in **Table 1** show that the serum IL-2 levels of cynomolgus monkeys treated with BPB-101 (30 mg/kg or 100 mg/kg) was approximately 1.5 pg/mL one day after the fifth administration, a value similar to the maximum value in the control group (one day after the third administration). Serum IL-6 levels in the treatment groups peaked 6 hours after the end of the first administration and then decreased 24 hours after the first dose. Although serum IL-6 levels subsequently increased again with increasing dose, they remained below the initial peak observed 6 hours after the first administration. The levels of TNF-α and IFN-γ in the serum of cynomolgus monkeys in all the groups were below the lower limit of detection of the assay kit at each time point. Typically, CRS is characterized by a rapid, significant increase in cytokine levels over a short timeframe, with levels that continue to increase and do not easily return to baseline (the cytokine level of cynomolgus monkey before drug injection). However, BPB-101 treatment did not enhance cytokine release. During the administration and recovery periods, all monkeys in the BPB-101 group maintained a good general condition with normal autonomic activity. Thus, BPB-101 displayed a favorable safety profile *in vivo*.

**Table 1:** Cytokine analysis at various time points following intravenous administration of different doses of BPB-101 in cynomolgus monkey.

IL-2 (pg/mL)	Control group		30 mg/kg group		100 mg/kg group	
	N	Mean ± SD	N	Mean ± SD	N	Mean ± SD
Before the first administration	1	1.097	/	/	/	/
2 hours after the end of the first administration	1	0.851	/	/	/	/
6 hours after the end of the first administration	1	0.844	/	/	/	/
24 hours after the end of the first administration	1	0.983	/	/	/	/
1 day after the third administration	1	1.305	2	0.846 ± 0.0813	/	/
1 day after the fifth administration	/	/	2	1.620 ± 0.6548	1	1.572
IL-6 (pg/mL)	Control group		30 mg/kg group		100 mg/kg group	
	N	Mean ± SD	N	Mean ± SD	N	Mean ± SD
Before the first administration	3	1.073 ± 0.6285	3	0.751 ± 0.4538	3	2.355 ± 1.5642
2 hours after the end of the first administration	5	3.191 ± 1.0260	5	5.220 ± 4.1426	5	3.524 ± 1.0796
6 hours after the end of the first administration	5	2.327 ± 1.2779	5	7.164 ± 7.4416	5	5.110 ± 2.9338
24 hours after the end of the first administration	4	1.373 ± 1.1042	5	1.060 ± 0.5945	4	1.795 ± 1.3313
1 day after the third administration	4	1.558 ± 1.6487	5	3.610 ± 3.3586	5	4.784 ± 3.8497
1 day after the fifth administration	2	0.818 ± 0.1732	5	5.886 ± 5.5312	5	4.693 ± 4.1139
TNF-α (pg/mL)	Control group		30 mg/kg group		100 mg/kg group	
	N	Mean ± SD	N	Mean ± SD	N	Mean ± SD
Before the first administration	/	/	/	/	/	/
2 hours after the end of the first administration	/	/	/	/	/	/
6 hours after the end of the first administration	/	/	/	/	/	/
24 hours after the end of the first administration	/	/	/	/	/	/
1 day after the third administration	/	/	/	/	/	/
1 day after the fifth administration	/	/	/	/	/	/
IFN-γ (pg/mL)	Control group		30 mg/kg group		100 mg/kg group	
	N	Mean ± SD	N	Mean ± SD	N	Mean ± SD
Before the first administration	/	/	/	/	/	/
2 hours after the end of the first administration	/	/	/	/	/	/
6 hours after the end of the first administration	/	/	/	/	/	/
24 hours after the end of the first administration	/	/	/	/	/	/
1 day after the third administration	/	/	/	/	/	/
1 day after the fifth administration	/	/	/	/	/	/

Cytokine indicators that fall below the lower limit of quantitation (BLQ) are not included in the statistical analysis, hence the number of samples included in the statistics is reduced. A '/' indicates that all data in the group were below the detection limit of the assay kit.

### BPB-101 exhibits an outstanding stability

**Figure 7:**
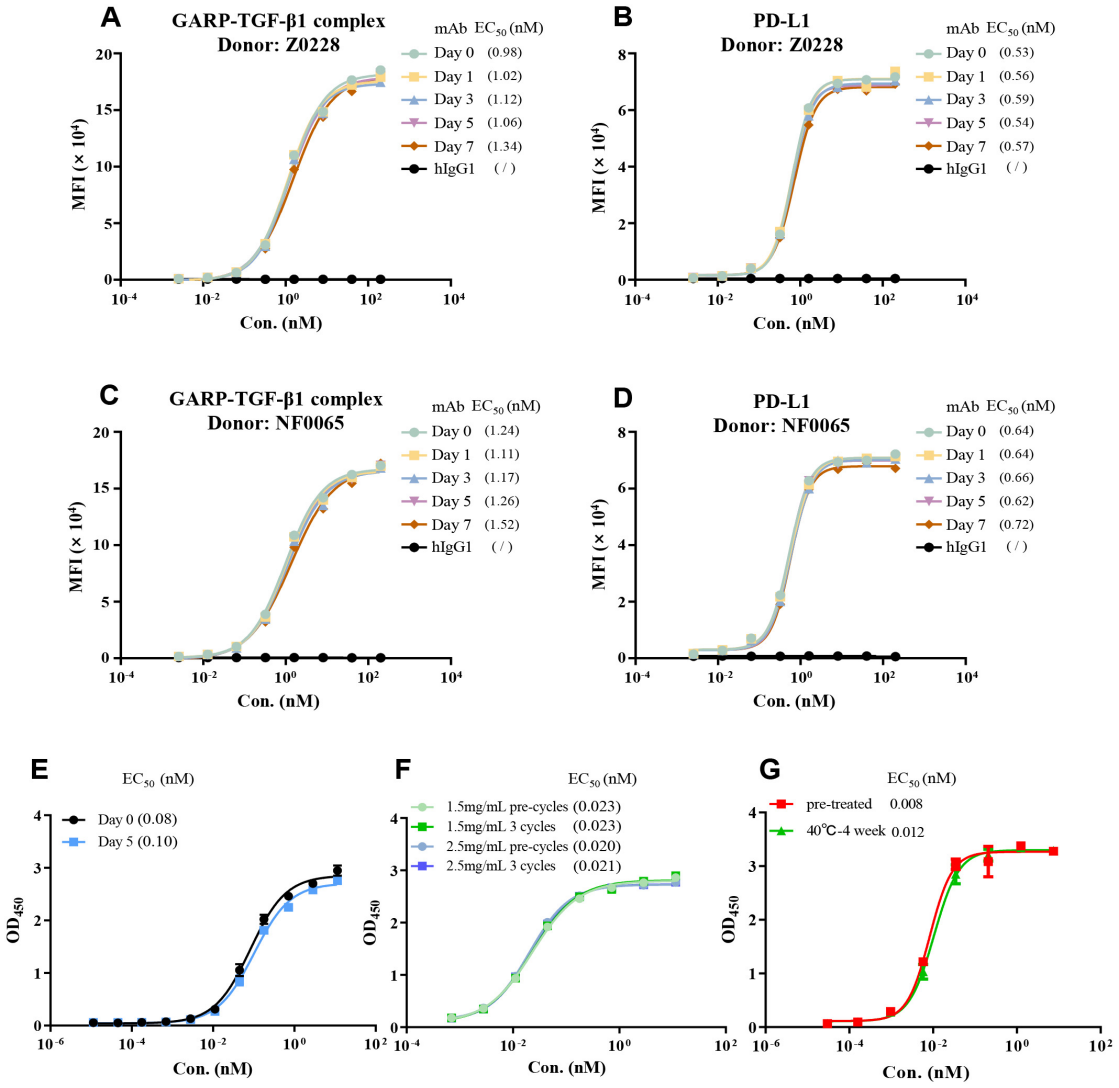
Evaluation of BPB-101 stability *in vitro*. **(A–D)** First, BPB-101 was incubated in a solution with a high concentration of serum (> 90%) at 37°C for 0, 1, 3, 5 or 7 days. Then, the ability of BPB-101 to bind to the GARP-TGF-β complex and PD-L1 was determined via FACS [**(A, B)** Donor: Z0228; **(C, D)** Donor: NF0065]. **(E)** The binding of BPB-101 to the GARP-TGF-β complex and PD-L1 after exposure to 5000 lux and 85 μW/cm^2^ light conditions for 0 and 5 days was determined by ELISA. BPB-101 was placed horizontally during the exposure period. **(F)** Binding of BPB-101 to human TGF-β1 and PD-L1 before and after freeze-thaw cycles, as determined by ELISA. **(G)** Binding of BPB-101 to the GARP-TGF-β complex and PD-L1 after incubation at 40°C for four weeks, as determined by ELISA.

Stability is one of the important factors that determines the biological function of antibodies. Here, we investigated the stability profile of BPB-101 in human serum. After incubating BPB-101 for one week in a solution containing a high concentration of serum (> 90%) at 37°C, it was still able to bind efficiently to GARP-TGF-β complex-expressing cells (**Figures 7A, C**), and PD-L1-expressing cells (**Figures 7B, D**), with little changes in the EC_50_ values as determined by FACS at different time points. Moreover, we conducted other stability experiments, including assessments after exposure to illumination for 5 days (**Figure 7E**), after repeated freezing and thawing cycles for 3 cycles (**Figure 7F**), and after high-temperature acceleration tests (40°C, 4 weeks) (**Figure 7G**), were completed. The EC_50_ values, as determined by ELISA, changed only slightly. All these results confirmed the excellent stability of BPB-101.

## Discussion

The evolution of tumor immunity has indeed unveiled treatment options for cancer patients, effectively prolonging survival and improving the quality of life. However, the fact that only a minority of patients respond to currently available therapies underscores the need for developing novel immune drugs. Within the TME, tumor cells orchestrate an environment conductive to their own growth and survival by directly or indirectly modulating various factors. TGF-β and the PD-1/PD-L1 pathways are two critical and well-studied mechanisms that suppress antitumor immunity in the TME. The development of antitumor drugs targeting the PD-1/PD-L1 axis has been relative success (42), with the launch of numerous products globally, including monoclonal antibodies against PD-1 or PD-L1, and bispecific antibodies targeting PD-1/CTLA-4 (43). Despite these advances, the development of therapeutics targeting TGF-β continue to encounter significant challenges (5).

TGF-β plays a dual role, not only regulating normal cell growth but also promoting tumor growth and metastasis in the advanced TME. It orchestrates tumor angiogenesis, facilitates tumor metastasis, and stimulates tumor fibroblast growth while simultaneously inhibiting the activities of various immune cells. Consequently, transforming the TME into an immune-supportive microenvironment that bolsters the antitumor functions of immune effector cells is crucial. Tumor cells, in addition to immune cells, also produce TGF-β. Thus, merely inhibiting or eliminating the active TGF-β present in the TME is insufficient, as tumor cells can continue to thrive due to the continuous secretion of TGF-β by themselves or by immune cells.

In light of these insights, we developed a tri-functional bispecific antibody, BPB-101. The Fab arm of BPB-101 specifically targets the GARP-TGF-β complex and/or the SLC, as well as active TGF-β. The C-terminus of BPB-101 incorporates an anti-PD-L1 nAb (VHH), which can effectively disrupt PD-1/PD-L1 signaling. This bispecific antibody is designed to reduce TGF-β levels in the TME and curb the immune evasion tactics of tumor cells. Therefore, BPB-101 has the potential to significantly reverse the immunosuppressive microenvironment and reactivate systemic antitumor immunity.

Upstream of the TGF-β axis, four monoclonal antibodies against GARP have been studied in clinical trials: ABBV-151 (AbbVie), SRK-181 [Scholar Rock Inc. (5)], DS-1055a [Daiichi Sankyo (44)], and HLX-60 (Henlius, NCT05606380). These drugs target hGARP, LAP-TGF-β or the hGARP-TGF-β complex, blocking only one of the TGF-β production pathways, and they cannot neutralize active TGF-β that is already present in the TME. Additionally, drugs developed to target TGF-β or TGF-βR, such as GC1008 (Genzyme, phase III), SAR439459 (Sanofi, phase III), NIS793 (Novartis, phase III), M7824 (Merck, phase II/III), and SHR-1701 (Hengrui, phase III), can only neutralize active TGF-β and cannot block TGF-β production.

Our BPB-101 fully addresses the shortcomings of the aforementioned drugs. The data showed that BPB-101 efficiently bound to its targets-GARP-TGF-β complex, active TGF-β and PD-L1-at both the cell and protein levels. However, DS-1055a, which targets GARP, and ABBV-151, which targets the GARP-TGF-β1 complex, both failed to bind to active human TGF-β1.

Interestingly, we did not detect the binding activity of ABBV-151 to the GARP-TGF-β1 complex by ELISA. However, M7824, which theoretically does not bind to the GARP-TGF-β complex, produced a binding curve to the GARP-TGF-β complex. This result probably occurred due to conformational changes that occurred when the GARP-TGF-β complex antigen was coated, as this phenomenon was not observed in the FACS analyses. These data also suggest that cellular-level detection is necessary to analyze antibody function.

To verify whether BPB-101 also associates with the SLC, we developed a BLI-based assay. The results showed that BPB-101 efficiently bound to the SLC, although the exact binding site remains unclear. In contrast, ABBV-151, M7824 and GC-1008 did not exhibit binding to the SLC. These findings suggest that BPB-101 has the potential to block the three active TGF-β release pathways: cleavage of the LAP domain, the SLC-LTBP complex, and the GARP-TGF-β complex (10). Therefore, this molecule is distinct from currently available GARP/TGF-β-targeting antibodies or fusion proteins. Consequently, BPB-101 may have the potential to address TGF-β immunosuppression in the TME, a challenge that has plagued the field for decades.

The anti-PD-L1 VHH (BPB-PD-L1), derived from an alpaca antibody library, is characterized by small size, high affinity and stable performance (45), which may contribute to improved CMC (Chemistry, Manufacturing and Controls) production and drug stability.

Studies have shown that Tregs inhibit immunity in response to the environment, particularly by promoting tumor growth through the release of TGF-β (46). Furthermore, we demonstrated the ability of our antibody to inhibit TGF-β1 secretion by human Tregs, suggesting its potential for clinical application.

Safety, effectiveness, and drug availability are important criteria for evaluating therapeutic drugs. We confirmed that BPB-101 did not cause significant cytokine release *in vitro*. Furthermore, an *in vivo* safety evaluation of BPB-101 was conducted in cynomolgus monkeys with single-dose and multiple-dose administrations. This evaluation focused on the levels of IL-2, IL-6, TNF-α and IFN-γ at different time points after administration. Since there is no defined threshold for each cytokine, CRS is usually assessed based on trends in cytokine expression. CRS is characterized by a rapid and significant increase in cytokine levels over a short period, with difficulty in reverting to normal levels. However, in our study, the cytokine levels were very low (TNF-α and IFN-γ), similar to those in the control group (IL-2), or initially increased and then decreased (IL-6); these results were inconsistent with the characteristics of CRS. Therefore, we concluded that BPB-101 treatment did not enhance cytokine release and displayed a favorable safety profile.

Given that there is no species crossover within the GARP and PD-L1 genes between mice and humans, we utilized MC38-hPD-L1 tumor cells and human GARP transgenic mice for further studies. First, we demonstrated that BPB-101 effectively distributed to the tumor within an hour and continued to accumulate at the tumor site over time. Then, we further investigated the antitumor efficacy of BPB-101 *in vivo*. The antitumor activity of BPB-101 was notably better than that of monoclonal antibodies alone or in combination. The tumor volume of the mice in the BPB-101 group gradually decreased (P < 0.001 vs. PBS) during treatment. Moreover, tumors were completely eliminated in 50% (4/8) of mice, indicating the antitumor potency of BPB-101.

It has been reported that the use of TGF-β inhibitors increases the risk of bleeding (47). Specifically, Mayur S. Mitra et al. revealed that treatment with anti-TGF-β neutralizing monoclonal antibodies, which block all three isoforms, was associated with an increased risk of bleeding and cardiac toxicity in mice and monkeys (48). In a phase I clinical study of M7824, the incidence of bleeding-related AEs was 39.3%, and the incidence of grade 3 and higher bleeding-related AEs was 10.2% (49, 50). However, bleeding was not observed in our *in vivo* preclinical study with cynomolgus monkeys, indicating that the safety profile of BPB-101 may differ from that of TGF-β inhibitors.

Moreover, stability assessments, including evaluation of stability in serum, after exposure to light, after repeated freeze-thaw cycles, and after high-temperature stress tests, confirmed the stability of BPB-101.

In summary, BPB-101 effectively blocks the immunosuppression of TGF-β by simultaneously targeting the source of TGF-β production and neutralizing active TGF-β. Through this effect, coupled with the inhibition of the PD-L1 axis, BPB-101 elicits potent antitumor activity in preclinical models. Given these very encouraging preclinical observations, a good safety profile, and druggability, BPB-101 has entered clinical development to examine its safety and preliminary efficacy in advanced-stage cancer patients. During the dose-escalation phase of the clinical trial, BPB-101 has shown good safety and has not induced CRS.

Research has shown that in patients with acute myeloid leukemia (AML), the expression of GARP on CD4^+^ T cells is elevated, leading to increased levels of TGF-β1. This increase inhibits the antitumor activity of NK cells, resulting in their dysfunction. Further studies indicate that blocking TGF-β1 signaling can enhance the anti-tumor activity of NK cells in leukemia xenograft mouse models (51). Therefore, we believe that BPB-101 not only has potential for the treatment of solid tumors but also holds promise for applications in hematological malignancies. We anticipate that BPB-101 will benefit more cancer patients and fill an important gap in currently available treatment options.

However, there are still limitations and areas for further exploration. The intricate relationships and synergies among the three functions of BPB-101, its interaction with immune cells, and its precise mechanisms of action remain to be fully elucidated. Future studies should focus on analyzing the characteristics of immune cells at different tumor stages, as well as exploring more indications and disease models. The selection of appropriate biomarkers will also be critical for advancing the clinical development of BPB-101.

Moreover, the exploration of combination therapies and the ability to address unmet clinical needs remain paramount. Despite the promising preclinical performance and safety profile of BPB-101, understanding its place within the broader landscape of cancer therapy, particularly in combination with existing treatments, will be essential for maximizing patient benefit.

## Data Availability

The original contributions presented in the study are included in the article/**Supplementary Material**. Further inquiries can be directed to the corresponding authors.
