# Supplementary material for: Discovery and preclinical evaluation of BPB-101: a novel triple functional bispecific antibody targeting GARP-TGF-β complex/SLC, free TGF-β and PD-L1
Source: Front Immunol. 2024 Nov 20;15:1479399. doi: 10.3389/fimmu.2024.1479399 (PMC11615479; doi:10.3389/fimmu.2024.1479399)
Supplement: Supplementary file 1 [file DataSheet1.pdf]

Supplementary Information for

Discovery and preclinical evaluation of BPB-101: a novel triple functional bispecific antibody targeting GARP-TGF-β complex/SLC, free TGF-β and PD-L1

Wenxin Xu[1,*], Jieying Xu[1], Pingcui Li[1], Deyu Xu[1], Hongjie Cheng[1], Huan Zheng[1], Li Zhang[1], Mengmeng Liu[1], Siyuan Ye[1], Mengshi Jiang[1], Wenqi Yu[1], Jiabing Wang[1], Lieming Ding[1,*]

Authors affiliations:

[1]The R&D department of Betta Biologic, Betta Pharmaceuticals Company, Ltd, 355 Xingzhong Road, Hangzhou, Zhejiang, 311100, China

***Corresponding authors:**

Wenxin Xu, Betta Pharmaceuticals Company, Ltd, 355 Xingzhong Road, Hangzhou, 311100, China. Email: wenxin.xu@bettapharma.com

Lieming Ding, Betta Pharmaceuticals Company, Ltd, 355 Xingzhong Road, Hangzhou, 311100, China. Email: lieming.ding@bettapharma.com

Supplementary Materials and Methods

Materials

Avelumab (MCE, HY-108703) and atezolizumab (MCE, HYP9004) was purchased from MCE (China) Medchemexpress Co., Ltd. hIgG1 (Biolegend, 403502), streptavidin-PE (Biolegend, 405204), CFSE cell division tracker kit (Biolegend, 79898), anti-human CD3 antibody (Biolegend, 317326), anti-human CD28 antibody (Biolegend, 302934), PerCP/Cyanine5.5 anti-human CD3 antibody (Biolegend, 317336), APC anti-human CD4 antibody (Biolegend, 317416), PE/Cyanine7 anti-human CD8 antibody (Biolegend, 344712), Brilliant Violet 510™ anti-human CD25 antibody (Biolegend, 302640), Brilliant Violet 421™ anti-human CD127 (IL-7Rα) antibody (Biolegend, 351310), APC anti-human HLA-DR antibody (Biolegend, 327022), PE/Cyanine7 anti-human CD123 antibody (Biolegend, 306010), PE/Cyanine5.5 anti-human CD14 antibody (Biolegend, 301824), Brilliant Violet 421™ anti-human CD16 antibody (Biolegend, 302038), Brilliant Violet 510™ anti-human CD56 (NCAM) antibody (Biolegend, 318340), APC anti-human CD19 antibody (Biolegend, 363006), FITC anti-human CD45 antibody (Biolgend, 304006) , Zombie NIR™ Fixable Viability Kit (Biolegend, 423106) and recombinant human M-CSF (Biolegend, 574806) was purchased from Biolegend (Beijing) Biotechnology Co., Ltd. Human PD-L1 protein (ACRO, PD1-H5229), human GARP-TGF-β1 complex (ARCO, GA1-H52W9), biotin-PD-L1 protein (ARCO, PDL-H82F2), CD80-mFc (ARCO, B71-H52A4) were purchased from ARCO Motor Industry Co., Ltd. DMEM (Gibco, 11995065), FBS (Gibco, 10099-141C), PBS (Gibco, 10010-023), Puromycin Dihydrochloride (Gibco, A1113803), Pen/Strep (Gibco, 15240-062) and hygromycin B (Invitrogen, 10687-010) were from Thermo Fisher Technology (China) Co., Ltd. anti-human IgG-Fc-HRP (Jackson, 109-035-098) and R-PE-conjugated AffiniPure Goat anti-human IgG (Jackson, 109-115-098) were from Jackson ImmunoResearch. G418 sulfate (108321-42-2) were from Sangon Biotech (Shanghai) Co., Ltd. Goat anti-human IgG-FITC (Abcam, 97224), R-PE-Goat anti-mouse IgG Fc (Abcam, 98742), APC-anti-CD14 (Abcam, ab9114) were from Abbot (Shanghai) Trading Co., Ltd. BSA (Life Science,

0332-100G) were from LifeScience Inc. Human IL-2 (200-02) and hIFN-γ antibody pair (BD, 555142) were purchased from Propetech and Becton, Dickinson Company, respectively. LDH detection kit (G1781), One GloLuciferase assay kit (E6120) and Cell-Titer Glo (G7572) were from Promega. BDTM Cytometric Bead Array (CBA) Human Th1/Th2 Cytokine Kit II (BD, 551809), anti-human CD28 (TGN1412, Abinvivo, B502901) and anti-human PD-L1 (Atezolizumab, Abinvivo, B2016) were bought from their respective agents. HRP-TGF-β1 antibody pair (NBP2-79297-5) were bought from Novus. Penicillin streptomycin solution (SV30010) was from Hyclone. Tween-20 (30189328) was bought from Sinopharm Group Co. Ltd. BPB-101, BPB-GARP, BPB-PD-L1, M7824 were all internally produced.

Cell lines and animals

293T-hPD-L1, 293T-hPD-1 and Jurkat-PD1-CD3zata-NFAT-Luc2 cells were purchased from KYinno Biotechnology Co., Ltd. 293T-hPD-L1 and 293T-hPD-1 cells were cultured with DMEM medium (10% FBS + 0.5 μg/mL puromycin). Jurkat-PD1-CD3zata-NFAT-Luc2 cells were cultured in complete RPMI 1640 medium (10% FBS + 100 μg/mL hygromycin + 1 μg/mL puromycin). 293F-GARP-TGF-β (4E9), 293-SBE-Res (1E9) and 293-TGF-β/GARP-αvβ6 (4D11) cells were obtained from Shanghai Chempartner Lifescience Co., Ltd. 293F-GARP-TGF-β (4E9) cells were cultured in DMEM (10% FBS + 0.5 μg/mL puromycin + 200 μg/mL G418). 293-TGF-β/GARP-αvβ6 (4D11) cells were cultured in DMEM (10% FBS + 2 μg/mL puromycin + 200 μg/mL G418. 293-SBE-Res (1E9) cells were cultured in DMEM medium (10% FBS + 200 μg/mL puromycin). PBMCs and Treg cells were purchased from AllCells/SAILY and cultured in complete RPMI 1640 medium (10% FBS + 1% Pen/Srep) and RPMI 1640 medium (10% FBS), respectively. All cells were cultured at 37°C in a humidified atmosphere containing 5% CO_2 (Thermo Fisher, Steri-cycle IL60). All cell lines were authenticated and not cultured for longer than 15 passages. Mycoplasma was screened by a PCR-based detection kit (Huabio, K0103) every one month.

Female and male MC38-hPD-L1 tumor-bearing mice and tumor-free mice were used at 8-10 weeks of age and were provided by GemPharmatech (Jiangsu) Co., Ltd. Experimental Animal Production License No.: SCXK (Su) 2018-0008, Experimental Animal Use License No.: SYXK (Su) 2018-0027, Experimental Animal Quality Certificate No.: 202262079. For antitumor experiment, C57BL/6-hGARP mice were used at 6-7 weeks of age and were provided by GemPharmatech (Jiangsu) Co., Ltd. Certificate number: 320727221100448943. Mice were bred and maintained under pathogen-free conditions. All experimental procedures were conducted according to protocols approved by the Jiangsu Provincial Department of Science and Technology Animal Control Committee (AP-MIJ220068).

Purify analysis of BPB-101

BPB-101, BPB-GARP and BPB-PD-L1 were expressed in Expi293F cells grown in shake flasks, and the cell culture supernatants were harvested by centrifugation. Then, the supernatants were passed over protein A agarose (MabSelect SuReTM from Cytiva). The bound antibodies were then washed with buffer consisting of 100 mM arginine (Sigma) and 150 mM NaCl (Jiangsu Province Qinfen Pharmaceutical Co., Ltd.) at pH 3.4. The CEX (cation exchange chromatography) was employed as a further purification step to remove aggregates and fragments. The proteins solution was adjusted to pH 5.0, and the CEX resin was equilibrated with 50 mM acetate (Sigma). The elution was conducted by a linear gradient from 0 to 500 mM NaCl plus 50 mM acetate, pH 5.0. The elution peak was collected in fractions, and samples were then analyzed for purity via SEC. Proteins were buffer exchanged into PBS buffer using Millipore Amicon ultracentrifuge tubes with a molecular weight (MW) cutoff of 30 kDa, and the protein concentrations were detected by Nanodrop One (Thermo Fisher).

Binding of BPB-101 to GARP-TGF-β complex, active TGF-β or PD-L1

Briefly, 293F-GARP-TGF-β (4E9) or 293T-hPD-L1 cells were plated in 96-well plates. Antibodies were added to the cells and incubated at 4°C (MeiLing, YC-968L) for 1 h.

Then, FITC-goat anti-human IgG was added to the cells, and the FITC fluorescence signal of the cells was analyzed by flow cytometry (Agilent, Advanteon).

Human PD-L1 protein, human GARP-TGF-β complex or human TGF-β protein was coated onto 96-well plates and cultured at 4°C overnight. After blocking with 2% BSA, antibodies were added and incubated for 2 h. Then, the plates were washed with PBST buffer three times (BioTek, 405LS). After adding goat anti-human IgG-Fc-HRP, the biotin signal was detected with a MiD5 strip reader (Molecular Devices, Spectra Max iD5) at 450 nm.

The dual binding capability of BPB-101 for the GARP-TGF-β complex and PD-L1 was also verified by flow cytometry. First, 293T-hPD-L1 cells and 293F-GARP-TGF-β (4E9) cells were labeled with the fluorochromes CSFE and FarRed, respectively. Then, the two cell lines and antibodies were cocultured at 4°C for 2 h, and flow cytometry was used to detect the double-positive cells.

Bilayer interferometry (BLI)

The binding kinetics were determined via an Octet RED96E system using anti-human Fc AHC sensors (Sartorius, 18-5064) or AHC2 sensors (Sartorius, 18-5142) to capture antibodies for 180 s at 10 μg/mL in PBS buffer. After a 120 s baseline step in SD buffer, 1:1 serial dilution (200 nM to 3.13 nM, plus 0 nM reference) in SD buffer of human antigens, including PD-L1 (Acro, PD1-H5229), GARP-TGF-β1 complex (Acro, GA1H52W9), LAP (Acro, LAP-H5213), and Latent-TGF-β1 (Acro, TG1-H524x), was performed for 400 s, followed by a 500 s dissociation step. The fitting of reference-subtracted data was performed in Octet Data Analysis 12.0 software, globally fitting all concentrations to a 1:1 binding model. Kinetic constants, including R^2, K_{on}, K_{off}, and K_D, were obtained from a set of association and dissociation curves constructed from a series of concentrations.

Competition binding assay of BPB-101 with PD-L1 and CD80

293T-hPD-1 and 293T-hPD-L1 cells were plated into 96-well plates (1E5/well) and

cultured with a mixture of antibodies with biotin-PD-L1 protein or CD80-mFc protein. One hour later, the cells were centrifuged (Eppendorf, 5810R) and washed twice with FACS buffer. After adding SA-PE or PE-labeled goat anti-mouse IgG Fc, cells were detected with flow cytometry.

Luciferase reporter gene assay (RGA)

To verify the blocking effect of BPB-101 on downstream of TGF-β and PD-L1 signalings, two reporter systems were utilized. 293T-hPD-L1 and 293-TGF-β/GARP-αvβ6 (4D11) cells were used as upstream cells and provided hPD-L1 or TGF-β protein, respectively. Jurkat-PD-1-CD3zeta-NFAT-Luc2 and 293-SBE-res (1E9) cells were used as effector cells to provide the fluorescent signals. Antibodies were diluted in gradients and added to 96-well plates containing upstream cells. Then, effector cells were added into plates and cocultured for 6 h. After adding One-Glo reagent, the fluorescence of effector cells was detected with a MiD5 strip reader (Molecular Devices, Spectra Max iD5).

Allogeneic mixed lymphocyte reaction experiment

PBMCs were resuspended in RPMI 1640 culture medium and adjusted to 1E6 cells/mL (BECKMAN, VI-CELL XR). Mature DCs from another donor were resuspended at 2E5 cells/mL. Then, 100 μL of PBMCs, 50 μL of DCs and 50 μL of antibody were mixed well in a 96-well plate and cultured at 37 °C for 5 days. The cells were then centrifuged, and the supernatant was collected for detection of the concentration of human IFN-γ with an ELISA detection kit according to the manufacturer's recommendations.

Cytokine secretion of Tregs

The anti-human CD3 and anti-human CD28 (1 μg/mL) were coated onto 96-well plates and incubated at 4 °C overnight. The plate was washed three times with DPBS, and Tregs cultured in RPMI 1640 containing 500 IU/mL recombinant human IL-2 were

added to the plate at 4E5 cells/well. Then, the cells were incubated with diluted antibodies at 37 °C for 5 days. The cells were then centrifuged, and the supernatants were collected for detection of the concentration of human TGF-β with an ELISA detection kit according to the manufacturer's recommendation.

Binding of BPB-101 to different immune cells in the blood

The red blood cells (RBCs) were removed from human blood samples with RBC lysis buffer (BioLegend, 420302). Then, FCR blocking reagent (Miltenyi, 130-059-901) was used to block nonspecific binding sites on immune cells. The serial dilution of BPB-101 and hIgG1 were cultured with the obtained PBMCs for 1 h at 4°C. Fluorescent antibodies to specifically label each kind of immune cell were added and incubated with cells in the dark for 30 min at 4°C. After washing twice with FACS buffer, cells were subjected to flow cytometry to analyze the binding of BPB-101 to $CD4^+$ T cells ($CD3^+CD4^+$), $CD8^+$ T cells ($CD3^+CD8^+$), Tregs ($CD3^+CD4^+CD25^+CD127^{low}$), pDCs (HLA-$DR^+CD123^+$), classical monocyte cells ($CD14^+CD16^-$), nonclassical monocyte cells ($CD16^+CD56^-CD14^-$), B cells ($CD19^+$), NK cells ($CD56^+CD16^+$) and NKT cells ($CD3^+CD56^+$).

Effect of BPB-101 on cytokine secretion by PBMCs

Freshly separated PBMCs from six donors were resuspended in RPMI1640 culture medium respectively and adjusted to 1E6/mL. BPB-101, anti-human PD-L1 and anti-human CD28 antibodies were coated onto 96-well plates and incubated at 4°C overnight. The plates were washed twice with culture medium to remove unbound antibodies. Then, PBMCs were plated into the plate and incubated in an incubator containing 5% CO_2 at 37°C for 2 days. The supernatant was collected, and the expression of IFN-γ, TNF, IL-2, IL-4, IL-6 and IL-10 was determined with ELISA kits according to the manufacturer's recommendations.

Biodistribution of BPB-101

The biodistribution of BPB-101 in MC38-hPD-L1 tumor-bearing mice was explored by the ^{89}Zr isotope labeling tracer method. First, BPB-101 was labeled with ^{89}Zr to obtain ^{89}Zr-BPB-101 for subsequent study. Then, four MC38-hPD-L1 tumor-bearing mice (Female-01, Female-02, Male-01, Male-02) and two healthy mice (Female, Male) were intravenously injected with 15 mg/kg ^{89}Zr-BPB-101. The radioactive dose was 100 μCi per mouse. PET/CT static scans were performed at 1 h, 8 h, 24 h, 72 h, 120 h, 168 h, 216 h and 336 h after administration. Radioactivity values per unit volume of brains, hearts, lungs, livers, kidneys, spleens, joints, muscles, tumors, tibias, bladders, thymuses, inguinals and lymph nodes were analyzed by PMOD software. The percentage injection dose per gram of tissue (%ID/g) was calculated according to the administration dosage.

The antitumor efficacy of BPB-101

The C57BL/6 mice were genetically engineered to highly express human GARP protein (C57BL/6-hGARP mice). MC38-hPD-L1 cells were collected, resuspended at 5E6/mL in DPBS, and inoculated subcutaneously into the C57BL/6-hGARP mice (100 μL per mouse). When the average tumor volume reached ~50 mm^3, the mice were randomly divided into four groups (n = 8). Mice were intraperitoneally injected with PBS, BPB-GARP (4.2 mg/kg), BPB-GARP + BPB-PD-L1 (4.2 + 2.3 mg/kg) or BPB-101 (5 mg/kg) twice per week for a total of four times. The changes of the tumor volume and body weight of the mice were recorded. At the end of the experiment, the mice were sacrificed, and the tumors were collected, weighed and photographed.

***In vivo* safety assessment in cynomolgus monkeys**

Cynomolgus monkeys were randomly divided into three groups, each group included 10 monkeys, half of which were male and half of which were female. The cynomolgus monkeys were intravenously injected with 30 mg/kg or 100 mg/kg BPB-101 (15 mL/kg), and cynomolgus monkeys that were injected with the same volume of solvent (15 mL/kg) were used as controls. BPB-101 or solvent was administered once a week

for 4 consecutive weeks (5 times in total). Then, the monkeys were allowed to recover from drug withdrawal for 4 weeks. The general condition of the monkeys was observed during the administration and recovery periods. Approximately 1 mL of blood was collected from the lower limb vein or other suitable parts of the monkeys at preset times: before the first administration, 2 hours after the end of the first administration, 6 hours after the end of the first administration, 24 hours after the end of the first administration, 1 day after the third administration and 1 day after the fifth administration. Then, the serum was separated by centrifugation (1800 × g, 10 min) at 15-25°C and stored below -66°C for analysis. The levels of IL-2, IL-6, IFN-γ (MSD, K156A0H-4) and TNF-α (MSD, K156UCK-4) were measured by electrochemiluminescence (ECL) according to the manufacturer's recommendations.

Evaluation of BPB-101 stability *in vitro*

Freshly separated human blood (Donors: Z0228 and NF0065) was centrifuged to obtain serum. BPB-101 was diluted to 400 nM in serum (serum > 90%). The diluted antibody diluent was divided into tubes and placed in an incubator at 37°C incubator. Samples were collected on days 0, 1, 3, 5, and 7, and the binding of BPB-101 to 293F-GARP-TGF-β or 293T-hPD-L1 was investigated via flow cytometry.

One milliliter of BPB-101 (25.7 mg/mL) was added to 2-mL vials, and the vials were sealed. The vials were placed horizontally and exposed to 5000 lux and 85 $\mu W/cm^2$ light for 5 days. Samples were collected before and after exposure and analyzed by dual-binding ELISA detection. BPB-101 was diluted to 2.5 mg/mL and 1.5 mg/mL with 0.9% NaCl solution and divided into microcentrifuge tubes. After being stored at -20°C for 3 hours, the BPB-101 samples were thawed at 2-8°C. This step was repeated three times (3 cycles). Samples were collected before and after freezing-thawing and analyzed by dual-binding ELISA. BPB-101 was divided into microcentrifuge tubes, and incubated at 40°C with 75% relative humidity for four weeks. Samples were collected before and after the high-temperature acceleration test and analyzed by dual-binding ELISA detection.

Supplementary Figures legends:

Supplementary Figure S1. The sequence analysis of BPB-101 with DS-1005a, ABBV-151, HLX-60 and GAPR-TGF-β inhibitor. Evolutionary tree analysis of VH (A) and VL (B) between BPB-101 and other antibodies analyzed by Uniprot-Align (http://www.uniprot org/align).

Supplementary Figure S2. The binding of BPB-101 to different antigens. The binding of BPB-101, BPB-GARP, M7824 or GC1008 to human TGF-β2 **(A)** or TGF-β3 **(B)** determined by ELISA. The EC_{50} values are indicated in the legend. **C,** The binding of BPB-101, ABBV-151 or DS-1005a to human TGF-β1 determined by ELISA. The EC_{50} values are indicated in the legends. The simultaneous binding of BPB-GARP, BPB-101, BPB-PD-L1 or hIgG1 to hPD-L1 and GARP-TGF-β complex at the concentrations of 100 nM , 20 nM, 4 nM, 0.16 nM, 0,032 nM, 0,0064 nM and 0.00128 nM was determined by FACS. The 293T-hPD-L1 cells and 293F-GARP-TGF-β (4E9) cells were labeled with CSFE ($FITC^+$) and FarRed (APC^+), respectively.

Supplementary Figure S3. Luminescence value of 293-SBE-RES cells in RGA, indicating the blocking effect of M7824, GC-1008 and hIgG1 to GARP-TGF-β signaling.

Supplementary Figure S4. Impact of antibodies on TGF-β1 secretion by Tregs. Concentrations of TGF-β1 in the supernatants of Tregs cocultured with BPB-PD-L1, ABBV-151, M7824, GC1008 or hIgG1 (12.5 nM, 50 nM or 200 nM) for 5 days were determined by ELISA. All the data are expressed as the mean ± SEM.

Supplementary Figure S5. Biodistribution of ^{89}Zr-BPB-101 in healthy C57BL/6-hGARP mice and the tumor volume changes of C57BL/6-hGARP mice bearing MC38-hPD-L1 tumors. **A**, The percentage injection dose per gram of tissue (%ID/g) of ^{89}Zr-BPB-101 in healthy C57BL/6-hGARP female and male mice at different time points.

B, Concentration of ^{89}Zr-BPB-101 in livers, spleens, kidneys, lungs and lymph nodes of mice at different time points was selectively shown. **C**, Tumor volume changes of tumor-bearing mice after injection of ^{89}Zr-BPB-101. All the data are expressed as the mean ± SEM.

Supplementary Figure S6. The antitumor effect of different antibodies. Original tumor images of mice in different groups at the end of the experiment (n = 8).

Supplementary Tables legends:

Supplementary Table S1. The sequence analysis of different antibodies. Comparison of VHH-CDR and VHH between BPB-101 with atezolizumab and M7824 carried out by Uniprot-Align (http://www.uniprot org/align).

Supplementary Table S2. The affinity of the GARP-TGF-β1 complex, SLC and PD-L1 toward immobilized mAbs and in different cases determined by BLI. Kinetics constants including, R^2, K_{on}, K_{off}, and K_D were obtained from a set of association and dissociation curves on a series of concentrations.

Supplementary Figure S1:

A

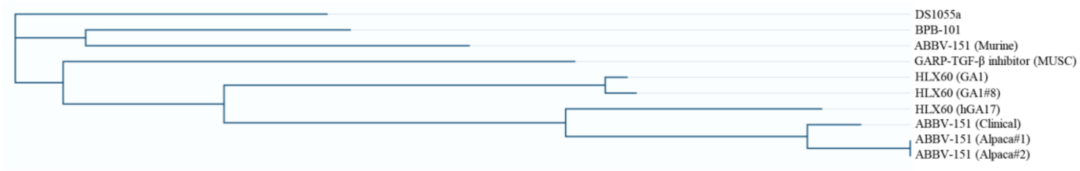

B

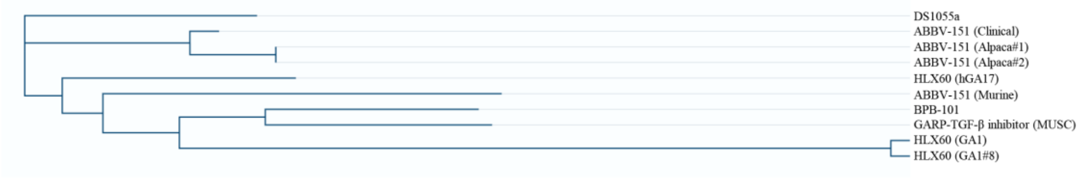

Supplementary Figure S2:

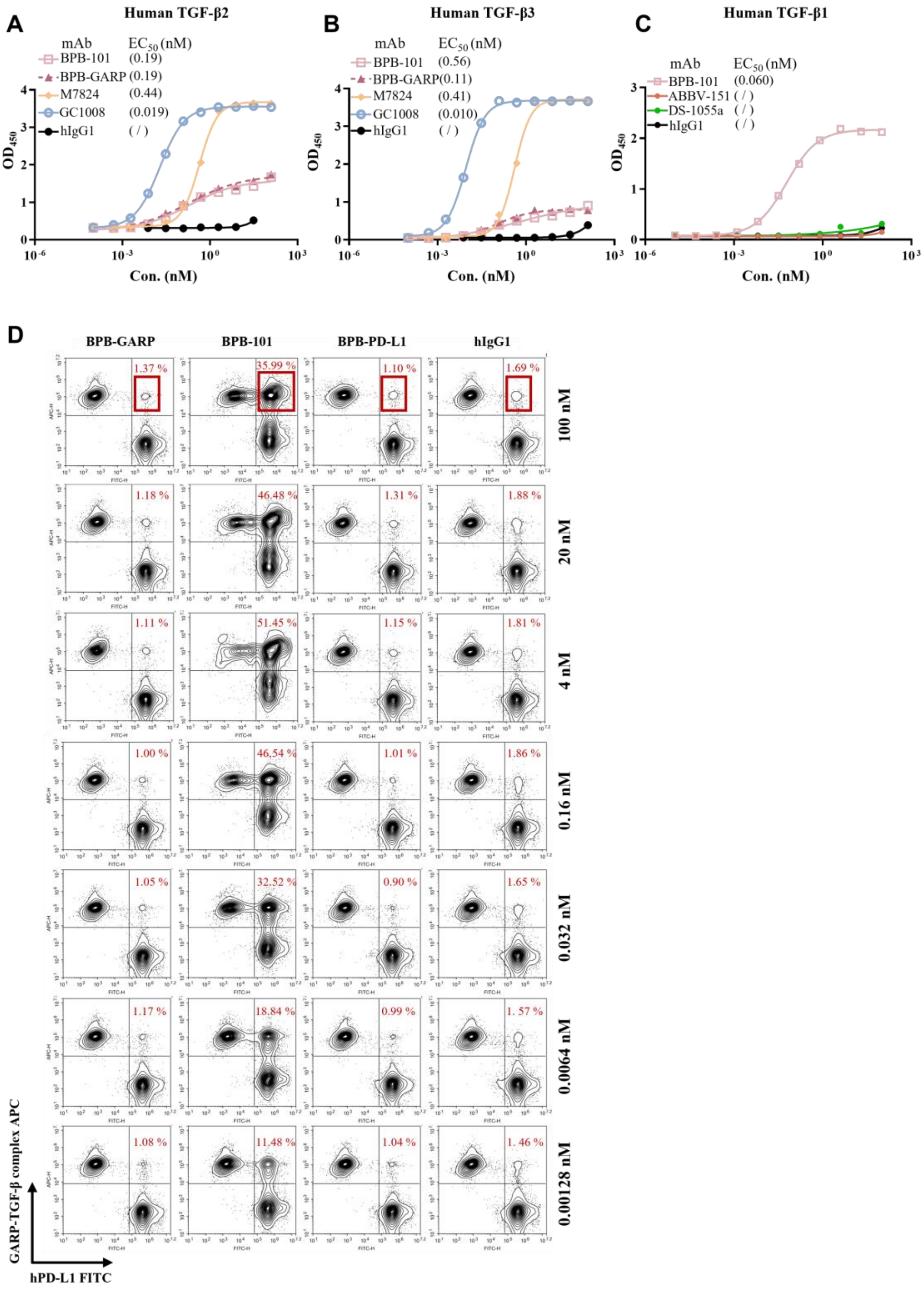

Supplementary Figure S3:

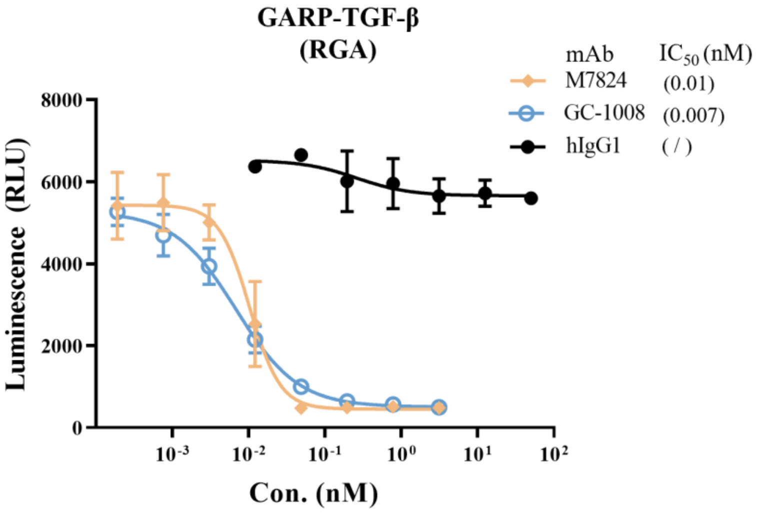

Supplementary Figure S4:

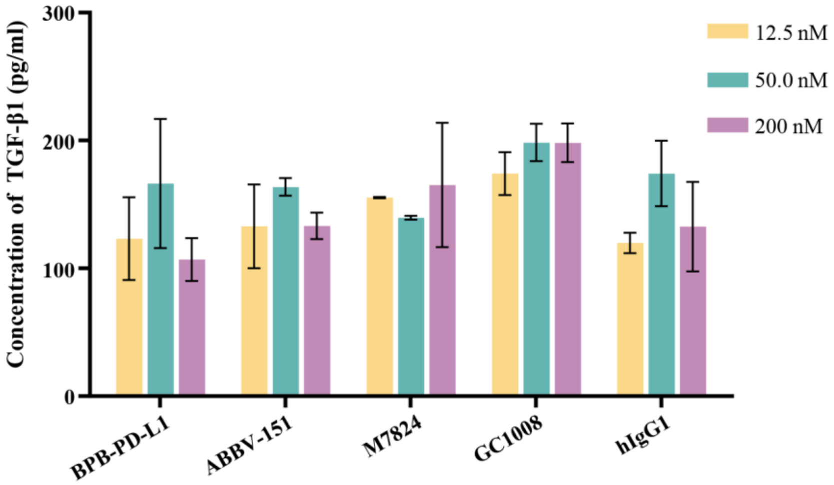

Supplementary Figure S5:

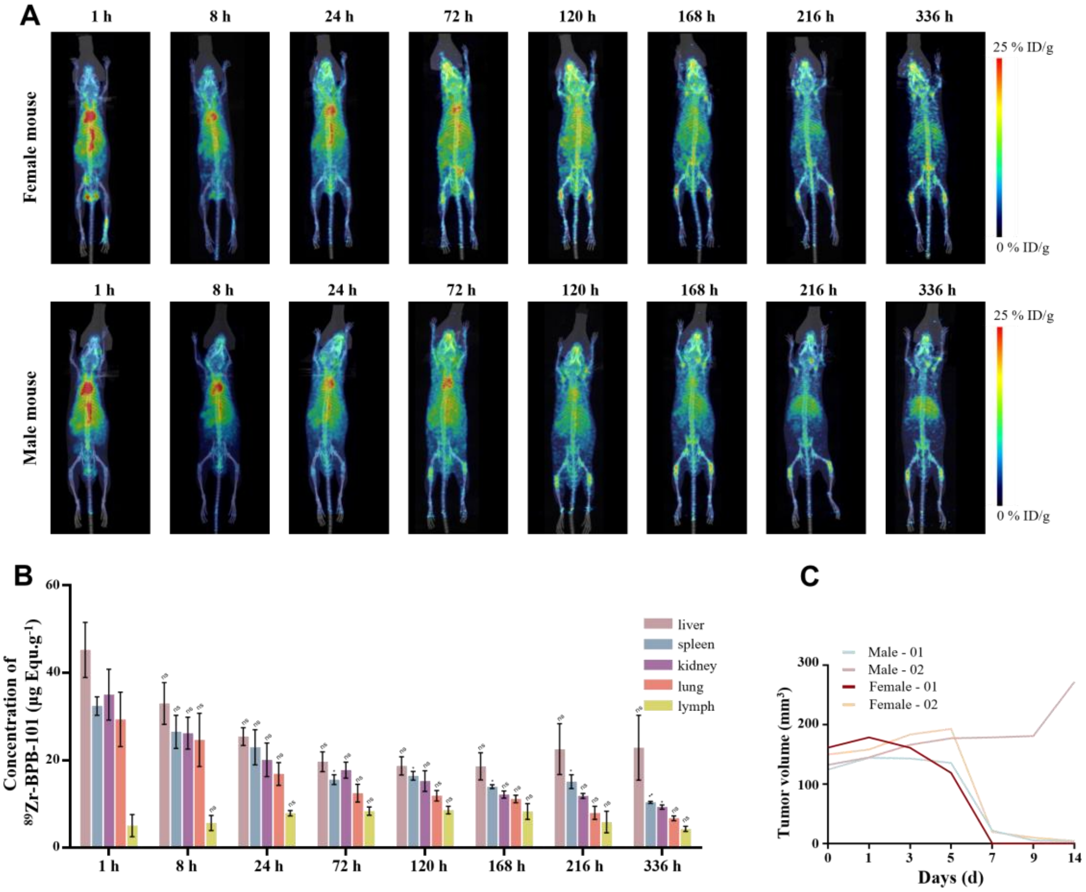

Supplementary Figure S6:

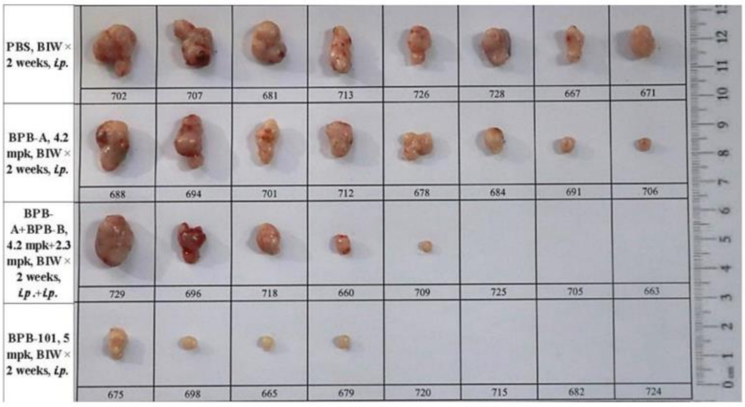

Supplementary Table S1:

VHH of BPB-101 compared with the VH of reference mAbs

Identity Per.(%)	BPB-101-VHH-CDR			BPB-101-VHH
	CDR-H1	CDR-H2	CDR-H3	
**Atezolizumab**	25.00%	37.50%	22.22%	72.88%
**M7824**	37.50%	33.33%	33.33%	72.50%

Supplementary Table S2:

Statistical data table of avidity of different antibodies to GARP-TGF-β1 complex, SLC and PD-L1

	Loading Sample ID	K_D (M)	k_{on}(1/Ms)	k_{off}(1/s)	R^2
Binding with GARP-TGF-β1 complex	BPB-101	$<1.0\times10^{-12}$	5.34×10^{5}	$<1.0\times10^{-7}$	0.9833
	ABBV-151	1.35×10^{-9}	2.27×10^{5}	2.82×10^{-4}	0.9909
Binding with Small Latent Complex	BPB-101	$<1.0\times10^{-12}$	1.71×10^{6}	$<1.0\times10^{-7}$	0.9970
	M7824	No binding			
	GC1008	No binding			
	ABBV-151	No binding			
Binding with PD-L1	BPB-101	4.96×10^{-10}	8.97×10^{5}	4.45×10^{-4}	0.9846
	M7824	1.31×10^{-9}	5.00×10^{5}	6.55×10^{-4}	0.9761
	Atezolizumab	1.28×10^{-9}	7.49×10^{5}	9.57×10^{-4}	0.9805
